# Wirelessly controlled modular automatic chambers for greenhouse gas flux monitoring in natural and agricultural ecosystems

**DOI:** 10.1016/j.ohx.2026.e00782

**Published:** 2026-04-30

**Authors:** Mikhail Mastepanov

**Affiliations:** a Department of Ecoscience, Aarhus University, Frederiksborgvej 399, 4000 Roskilde, Denmark; b Oulanka Research Station, University of Oulu, 93900 Kuusamo, Finland; c Division of Environment and Natural Resources, Norwegian Institute of Bioeconomy Research, Høgskoleveien 8, 1433 Ås, Norway

**Keywords:** Greenhouse gas, Flux, Chamber method, Automatic chamber, Wireless

## Abstract

The closed chamber method is widely used for measuring greenhouse gas fluxes (CO_2_, CH_4_, N_2_O) in natural and agricultural ecosystems. Automatic chambers are essential for long-term monitoring with high temporal resolution, but their production typically demands significant time, labor and expertise. While ready-to-use commercial solutions are available, many projects avoid them because of their high prices.

We present a cost-effective and scalable alternative: modular automatic chambers built from off-the-shelf components. These chambers feature integrated valves and wireless controllers, enabling flexible deployment without the need for multiplexers. Systems can be easily expanded by adding more units.

Our modular chambers have been successfully deployed in Arctic and subarctic field studies: north-eastern Greenland, natural wet tundra, two sites, 5 + 5 chambers, three summer seasons, CO_2_ and CH_4_ flux monitoring; northern Finland, natural boreal fen, 2–12 chambers, year-round measurements over four years, CO_2_ and CH_4_ fluxes; northern Norway, cultivated drained peatland, 30 chambers along a 300 m transect, four growing seasons (May–November), CO_2_, CH_4_, and N_2_O fluxes.

Across all sites, the chambers demonstrated reliability, ease of construction, operation and maintenance. While further improvements are always possible, the current design offers a practical and accessible solution for the broader scientific community.

## Graphical abstract

**Figure:**
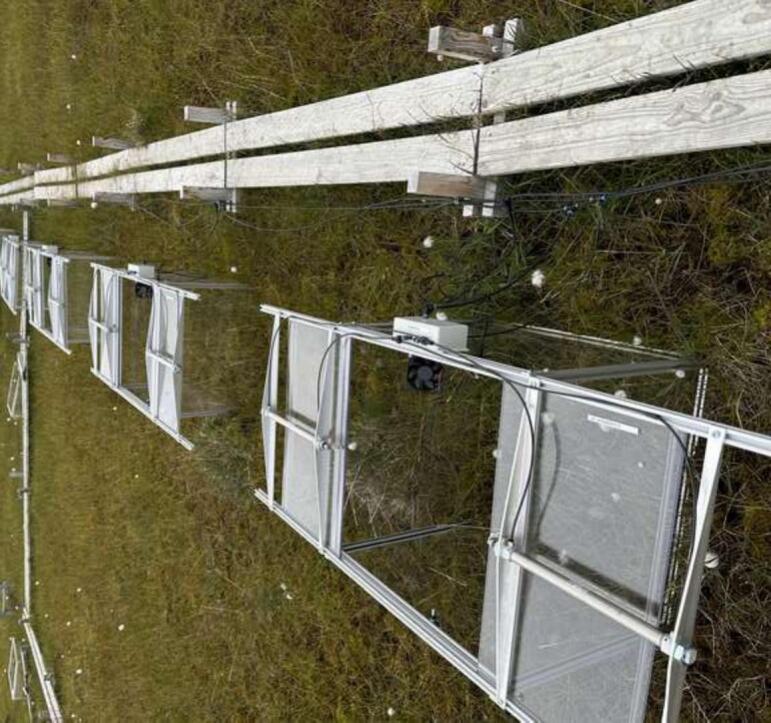

Specifications table

**Table:** 

Hardware name	• Automatic chambers for greenhouse gas flux measurements
Subject area	• Environmental, planetary and agricultural sciences • Educational tools and open source alternatives to existing infrastructure
Hardware type	• Field measurements and sensors
Closest commercial analog	*Li-Cor 8100*-*104/104C, 8200*-*104/104C, 8200*-*105/105C* *(Li-Cor, Lincoln, NE, USA)* *Eosense eosAC-T/O, eosAC-LT/LO* *(Eosense Inc., Dartmouth, NS, Canada)*
Open source license	*CC BY-NC*
Cost of hardware	*800 EUR per chamber*
Source file repository	http://doi.org/10.17632/35sc3vnrtg.2

## 1 Hardware in context

The closed chamber method is one of the most widely used techniques for measuring greenhouse gas (GHG) fluxes, such as CO_2_, CH_4_, and N_2_O, from natural and agricultural ecosystems [1]. Simple manual chambers are usually constructed by individual research groups, being relatively simple and inexpensive. However, manual chamber measurements are labor-intensive and limited in temporal resolution and consistency.

Automatic chambers enable high-frequency, long-term flux monitoring, producing detailed datasets that are critical for understanding ecosystem dynamics. Several research groups developed and used different models of automatic chambers starting from 1990s [2], [3], [4]. The use of such self-made automatic chambers has steadily increased over the years [5], [6], [7], [8], [9], [10], [11]. These chambers require substantial time and engineering expertise to produce, and often need experience to operate, service and repair.

Several commercially available automatic chamber solutions are offered nowadays, for example Li-Cor 8200-105/105C, 8200-104/104C and Eosense eosAC-T/O, eosAC-LT/LO. Such chambers are well designed, robust and user-friendly, but quite expensive (in the order of €10,000 per chamber). They are available in very few fixed sizes. Integrating several chambers to use with one gas analyzer requires additional costly multiplexers.

Here we describe a solution based on modular automatic chambers, which can be easily constructed from off-the-shelf components and cost less than one-tenth the price of commercial systems. These chambers are easy to assemble, deploy and repair when necessary. Some features, for example wireless control, scalable sizing, and chainable gas line connections are not currently available in commercial products.

Our chambers have been successfully applied in a few field studies in Arctic and subarctic ecosystems. At positive temperatures a set of these chambers can work unattended for weeks, during winter regular maintenance is required. This open-source solution provides a practical and accessible tool for researchers conducting GHG flux measurements in remote and challenging environments.

## 2 Hardware description

**Fig. 1:**
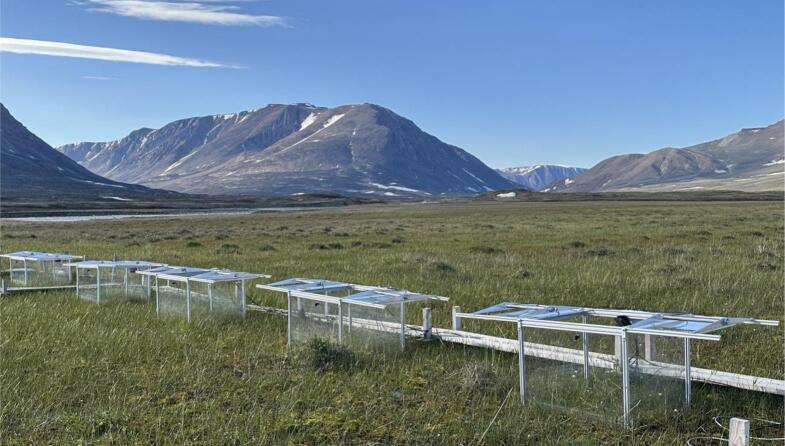
Modular automatic chambers in Zackenberg, NE Greenland.

**Fig. 2:**
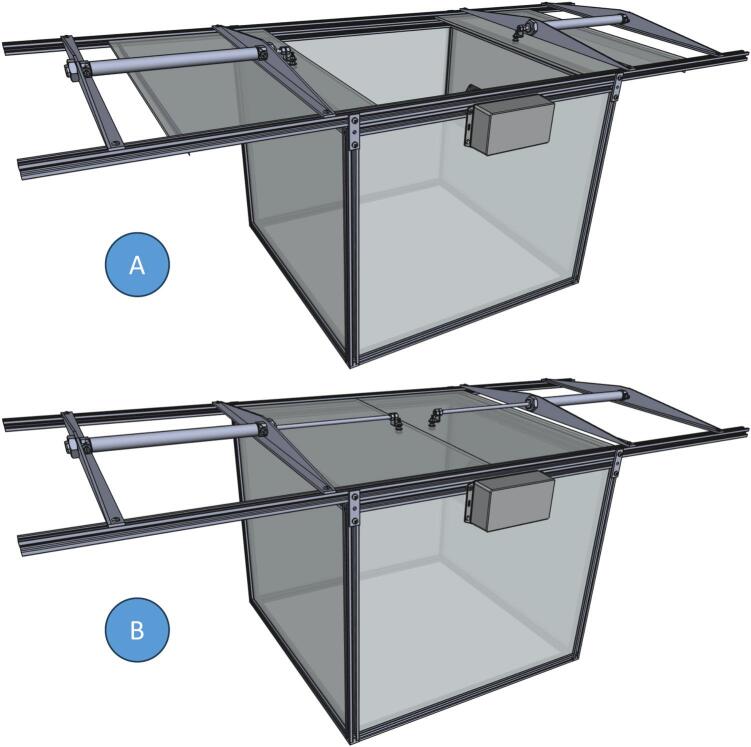
Screenshots of the chamber CAD model. A: chamber is open; B: chamber is closed.

The presented setup (Fig. 1) supports virtually any number of automatic chambers (from 2 to 30 in different projects we participated in), depending on the study site and research objectives. Each chamber (Fig. 2) is a box without bottom, with its top open between the measurements (Fig. 2A) and closed during the measurements (Fig. 2B). This box is put on the ground (with or without additional frame) to study gas exchange between this ecosystem and atmosphere. When the lid is open, gas concentrations inside the chamber are equal to atmospheric; when closed, the air inside the chamber is isolated from the ambient. In case of a positive net flux (emission) from the ecosystem to atmosphere, the concentration of this component inside the chamber increases; in case of negative net flux (fixation), the concentration decreases. The gas flux is calculated from this concentration change over time per covered area. To measure gas concentrations inside the chamber, a tube connects the chamber headspace with a gas analyzer. Equal amount of ambient air is replacing the sample air via a vent. This method is known as flow-through non-steady-state chamber technique [12]. Concentration change of each greenhouse gas in the headspace of the chamber can be used for calculation of its flux from between the ecosystem and atmosphere according to the following basic equation [13]:

(1) $$F_{\left(t\right)}=\frac{\mathrm{dc}}{\mathrm{dt}}\left(t\right)\frac{V}{A},$$

where $\frac{\mathrm{dc}}{\mathrm{dt}}\left(t\right)$ is concentration change with time, V is chamber volume, and A is chamber area.

There are different methods of flux calculation (e.g. linear regression, nonlinear exponential model, non-steady-state diffusive flux estimator model, etc. – see [14], [15], [16], [17]). The choice of flux calculation method is usually determined by ecosystem properties (e.g. porosity of the soil) and study aims, rather than chamber construction. Our chambers do not imply any limit on using any flux calculation methods; in our studies we mainly use the simplest linear method.

While chamber dimensions can be easily adjusted, this description focuses on our standard design with a footprint of 60 × 60 cm and a height of approximately 50 cm. Each chamber consists of:

• An aluminum frame made of V-Slot 2020 profiles
• Polycarbonate walls and lid composed of two sliding leaves driven by pneumatic cylinders
• A control box housing pneumatic and electronic components

Detailed construction instructions and sourcing information are provided in design files D01–D12. Each component has its own numerical ID (e.g. **1.01**), which will be used throughout the following text and all supplementary materials. CAD model of a chamber (main structural components only) is provided in supplementary materials, along with a video of this model in action.

### 2.1 Frame and structural components

The frame is assembled from Aluminum profiles **1.01**, **1.02** and **2.01** (V-Slot 2020 standard, can be ordered pre-cut in pieces of necessary length). Both the profiles and compatible connectors are available for purchase from various suppliers in different countries. Rectangular polycarbonate sheets **1.05** and **2.02** can also be ordered pre-cut to specified dimensions. Most of the small components of construction are sourced from RS Online – a company represented in many countries.

Pneumatic cylinders **2.05** are also available off-the-shelf; we used 20 mm diameter, 250 mm stroke model SMC CD85N20-250C-B (ISO 6432 standard). These cylinders have good resistance to dust and moisture, and feature an air cushion, so when the chamber closes or opens, the last few millimeters go extra smoothly. For chambers of different sizes cylinders with different stroke lengths can be used.

For cylinder mounts **2.03** we suggest two design solutions: simple (pre-cut L-shape aluminum profile with three holes) and elegant (the same, cut diagonally). Functionally both are the same, but the elegant mount is slightly lighter (see design sheet D04). In our chambers we used elegant mounts, produced for us by Ingemann Maskinfabrik A/S (Denmark). Simple design mount can be produced in place and reduces the chamber cost by approximately 100 Euro.

### 2.2 Modular construction

The lid and body are constructed as separate modules, connected via joining plates **2.14**. This modularity allows easy removal of the lid (e.g., for winter), or extension of the chamber height. The lid can be detached by removing four screws **2.15** (see design sheet D05).

Constructing the chambers of different heights is as easy as ordering aluminum frames **1.02** and polycarbonate sheets **1.05** in different sizes. Achieving different chamber area needs choosing the most suitable size of pneumatic cylinders, and recalculating sizes of lid components.

In our projects we always used transparent chambers; if opaque option is needed, polycarbonate could be replaced by opaque material (e.g. aluminum).

The modular design facilitates easy repair and maintenance. We recommend keeping a stock of spare parts and tools on-site.

**Fig. 3:**
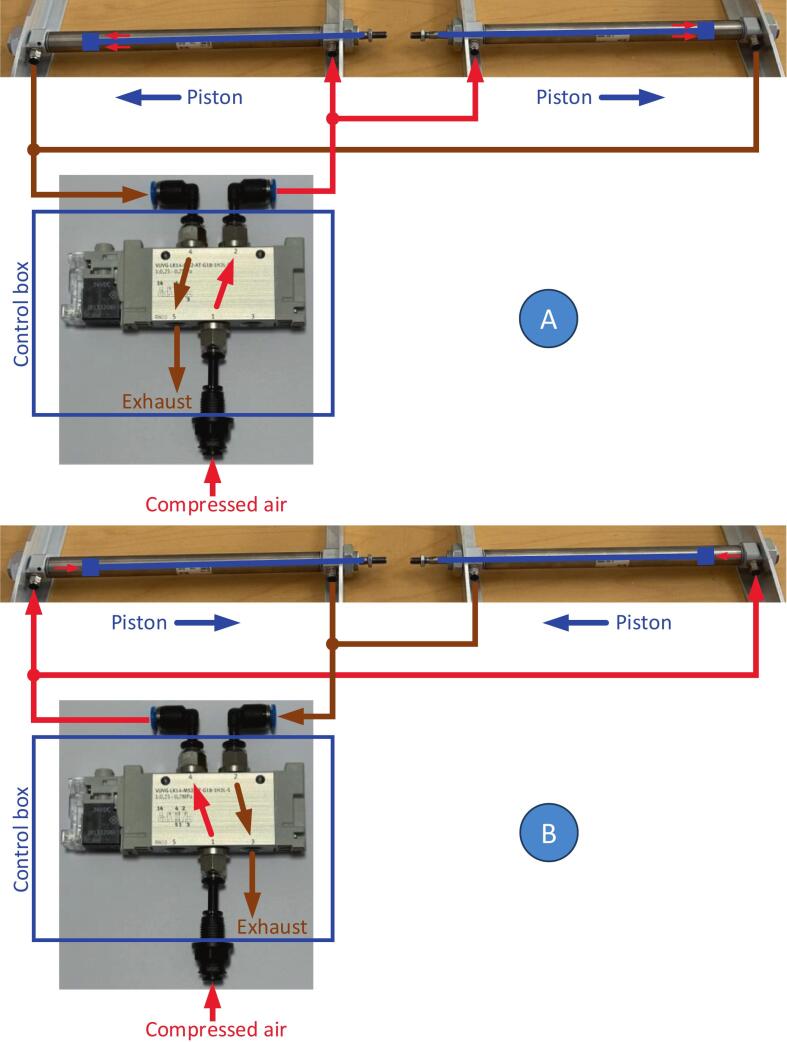
Pneumatic diagram. A: default state (unpowered compressed air valve), chamber opens and keeps open; B: powered compressed air valve, chamber closes and keeps closed.

The body and the lid together can be used as an automatic chamber, operated by an external source of compressed air (compressor, pressure regulator and valve manifold). When pressure is applied to the outer ports of the pneumatic cylinders, the chamber closes; when pressure is applied to the inner ports, it opens (see Fig. 3). However, the full potential of the construction is unlocked if each chamber is equipped with its own chamber control box.

### 2.3 Control box

Each chamber can be equipped with a dedicated control box (see design sheets D07 – D10), housed in a small plastic enclosure. It contains two solenoid valves (**3.03** for sample air, **3.07** for compressed air), and WiFi relay board **3.02** to operate these valves. Eight small-diameter holes should be drilled in the enclosure **3.01** according to the design sheet D10, then all components can be mounted inside. It can be recommended to assemble control boxes in stationary conditions (a lab or workshop), and produce one or two spares, so in case of any problems with control boxes in the field they can be replaced within few minutes and further repaired in stationary conditions. All the other parts can be transported to the field in a compact form, and chambers can be mounted in field conditions.

### 2.4 Pneumatically driven operation

The compressed air valve **3.07** is located in the control box of each chamber and should be permanently connected to a source of compressed air (1…3 bars). When the valve is unpowered (Fig. 3A), compressed air is directed to the inner ports of the pneumatic cylinders **2.05** causing retraction of the pistons and opening the chamber. When the chamber is fully open, it stays so without any further use of compressed air. When the valve **3.07** gets powered, it switches to the alternative position (Fig. 3B), directing compressed air to the outer ports of the pneumatic cylinders **2.05**, causing advancing of the pistons and closing the chamber. During the piston movement the air from the opposite part of the cylinder is exhausting via the free port of the valve. This exhaust is the only loss of the compressed air, implying no leaks in pneumatic gas lines. As the gas line between the valve **3.07** and pneumatic cylinders has a small volume (50–80 cm of 2 mm i.d. tubing), the total compressed air consumption is only about 0.6 standard liters per one open-close cycle at 2 bar pressure in the compressed air line.

### 2.5 Gas line configuration

In typical GHG studies multiple automatic chambers are connected to one or more gas analyzers via tubing (sample gas line). A common setup uses two tubes per chamber: one to transport sample air from the chamber to the analyzer, the other – to return it back to the chamber. This “air return” setup serves two purposes: keeping a closed air loop between the chamber and the analyzer, so the concentration in the chamber can be referred to the concentration in the analyzer; keeping the stable pressure in the chamber, as inflow equals to outflow. However, long tubing introduces lag time; for example, if the flow rate is 250 sccm (Li-7810), distance is 20 m and tube internal diameter is 4 mm, it takes about one minute for air to reach the analyzer, and one more minute to return to the chamber. Thus, for the first two minutes of measurement the ambient air is returned to the chamber, which is virtually the same as if the chamber had a vent and ambient air was entering the chamber.

**Fig. 4:**
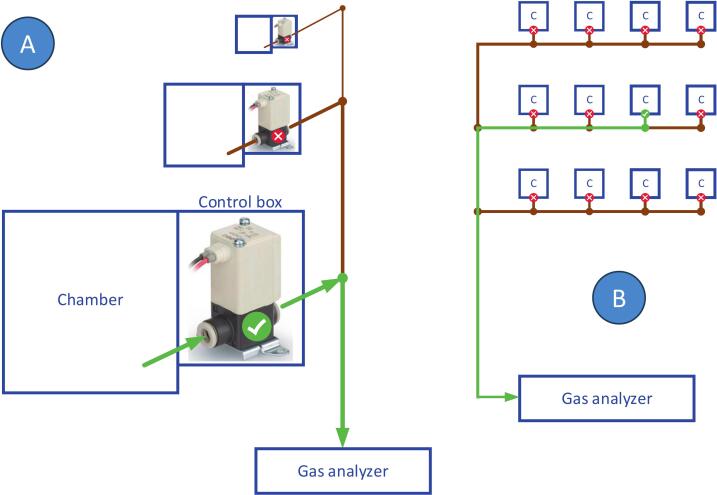
Chainable gas line diagram. A: sample air valve (inside the control box) of one chamber is powered, air from this chamber headspace is taken to the gas analyzer (green line). B: Gas line tubing can be chained in any configuration; only air from one chamber at a time comes to the analyzer.

**Fig. 5:**
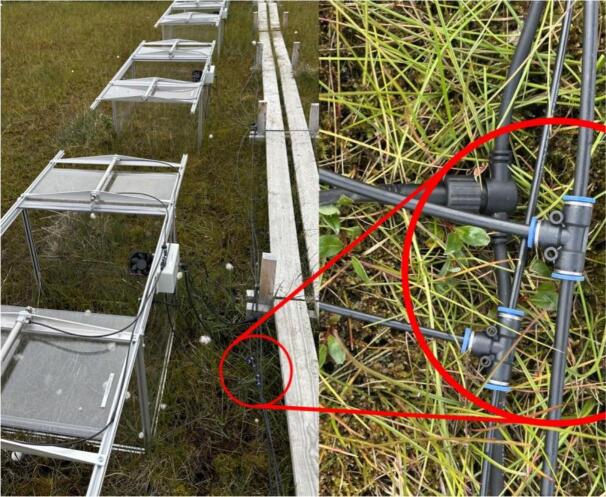
T-connectors for power, pneumatic and sample air tubing near each chamber (Zackenberg).

In our setup we decided to implement a vented chamber design and apply dilution correction (usually below 1% of flux value) during flux calculations. Thus, every chamber needs only one gas line tube, from the chamber to the analyzer. As the solenoid valve **3.03** is located in the beginning of this gas line (in the control box), the gas lines for individual chambers can be combined (Fig. 4). No multiplexer is needed at the analyzer side – all gas lines from individual chambers can be joined by simple T-connectors. This solution minimizes amount of tubing at the site, reducing the cost and simplifying troubleshooting. Evidently, pneumatic gas lines and power cables can also be chained the same way (Fig. 5).

### 2.6 Vent design, dilution and leaks

Construction of vents to compensate pressure changes in the chambers can be sophisticated [18], [19]. Surprisingly, we found that a simple vent made from 6 mm elbow adaptor (see D12) performs adequately in our field conditions. The sliding lid causes minimal pressure changes, and no significant artifacts were observed in concentration data.

As the ambient air enters the chamber through the vent during the measurement, it slightly dilutes the chamber headspace air and causes non-linear pattern in concentration during chamber closure. This dilution depends on the sample air flow rate (determined by a gas analyzer) and the effective volume of the chamber. A flux value, calculated upon a linear slope fit in case of dilution, can be corrected:

(2) $$F_{c}=F_{m}\left(1+\beta \right),$$

where $F_{m}$ is the gas flux, calculated upon a linear slope, $F_{c}$ is the corrected gas flux (both in the same units, i.e. mg m^−2^ h^−1^), and β is the correction factor (unitless). The simplified formula for this correction factor is:

(3) $$\beta \approx \left(t_{1}-t_{0}\right)\frac{Q}{2V},$$

where $t_{0}$ is the moment when chamber has closed (may or may not be the start of the slope used for flux calculation); $t_{1}$ is the moment chosen as the end of the flux calculation slope (may or may not be the moment when the chamber opens); Q is the sample gas flow rate, from chamber to analyzer (the same is the flow rate of ambient air into the chamber); V is the chamber volume. The units can be e.g. minutes for $t_{0}$ and $t_{1}$, liters per minute for Q, and liters for V, or any other, corresponding to each other, so β is unitless.

Although Eqs. (2), (3) are not a mathematically correct solution to the dilution differential equation, they give a reasonably accurate result. As an example, for our chamber volume 180 l, flow rate 0.25 l/min and 10 min slope starting at chamber closing moment, measured flux $F_{m}$ without correction is about 0.7% lower than a theoretically calculated flux value, while the corrected flux $F_{c}$ is only about 0.0008% lower. A theoretical background for applicability of linear flux calculations to flow-through chamber measurements with explanations of Eqs. (2), (3) is presented in Supplementary Materials 1; numerical evaluation of these calculations is presented in Supplementary Materials 2.

Small leaks through not perfectly airtight connections between chamber components do not influence the measurement results, as they act similar to the vent: ambient air is coming to the chamber. Only if the leaks are big, and air can both enter and leave the chamber (e.g. during strong wind), the dilution becomes stronger than via the vent. In this case the curvature of the concentration pattern is higher and is dependent on wind speed.

**Fig. 6:**
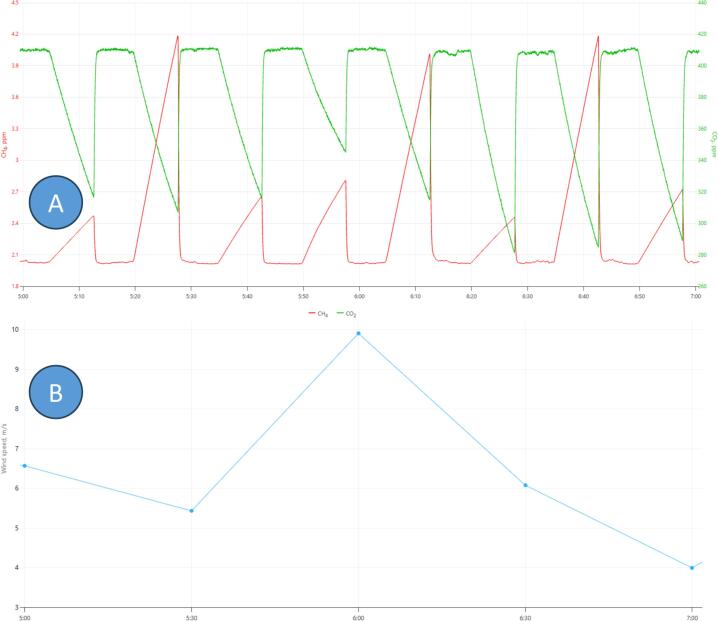
Example of data, collected by modular chamber setup (Zackenberg, NE Greenland) on a windy day. A: raw concentration data, CH_4_ (ppm) and CO_2_ (ppm), Li-7810; B: half-hourly average wind speed at the site, 2 m altitude, m/s.

An example of raw data (concentrations of CH_4_ and CO_2_) for two hours of measurements (site Zackenberg I) is presented in Fig. 6A, together with wind speed data from this site (Fig. 6B). The concentration pattern is typical for such chamber measurements: when the active chamber is open, the concentration of both gases is close to ambient; when chamber closes, concentration of CH_4_ increases (net emission), and concentration of CO_2_ is decreasing (net fixation). Two hours of measurements (Fig. 6A) represent 8 individual chamber measurements (5 chambers, 1.6 cycles). Concentration changes are not fully linear because of dilution effect of the vents; however calculated fluxes can be corrected using equations (2), (3). Individual chambers have different correction factors due to difference in volume; change of wind speed has a neglectable effect on the dilution.

### 2.7 Balanced weight distribution

**Fig. 7:**
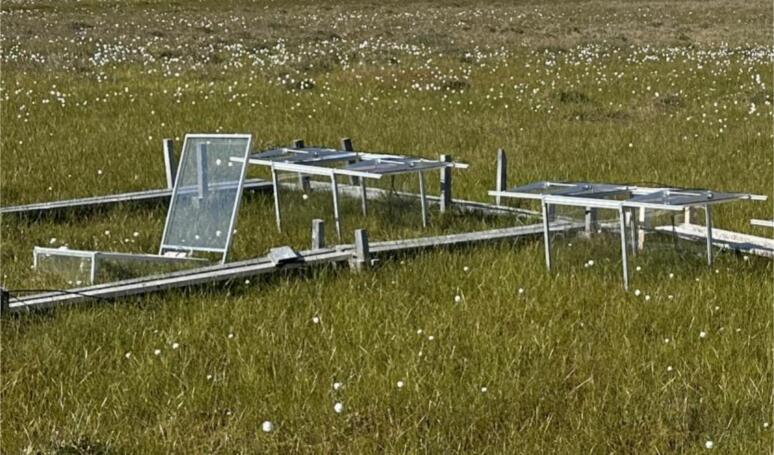
Asymmetrical chamber tilted over the years, versus symmetrical chambers (Zackenberg).

From long-term experience using automatic chambers in wetland ecosystems [9], [20] we observed that chambers, installed on a soft ground, tend to tilt with time, if their mass is not centered and/or if opening/closing momentum pushes them aside (Fig. 7). Developing new chambers, we tried to make them symmetrical around their center of mass, and construct the lid out of two equal leaves, moving inwards and outwards synchronously. As a result, our chambers can gradually sink in soft ground, but will not tilt.

Vertically opening lids have been observed vulnerable to strong winds. Strong gusts either close chambers, causing artificial conditions for vegetation, or break lid hinges. Chambers with horizontally sliding lids have been proven to work smoothly even at high wind speeds.

### 2.8 Wireless control

Although WiFi control of automatic chambers may seem less reliable than cable connection, it offers significant advantages for large-scale deployments. WiFi relay board **3.02** is installed to the control box of every chamber, and used to control both solenoid valves (**3.03** and **3.07**). Stable WiFi signal should be provided to cover every chamber, and each board 3.02 should be pre-configured according to the WiFi settings (see Build instructions for more details). Commands to open/close chambers can be sent from any networked device (e.g., computer, datalogger, Arduino). If a chamber fails to respond, the system can skip it and proceed with others. We have used WiFi-controlled chambers for extended periods across multiple sites with consistently high reliability.

### 2.9 Key advantages of new chambers

• Low cost compared to commercial alternatives
• Simplified assembly compared to custom-built solutions
• Availability and low cost of spare parts, easy field repair
• Adjustable chamber size
• Low energy consumption, possibility of autonomous powering
• No limit on number of chambers in setup, no need for multiplexers
• Minimal amount of tubing and cabling
• WiFi control from any networked device
• Proven performance in Arctic and subarctic ecosystems

## 3 Design files summary

The design files can be found at: http://doi.org/10.17632/35sc3vnrtg.2.

**Table:** 

**Design file name**	**Contents**	**File type**	**Open source license**	**Location of the file**
*D01*	General appearance	*PDF*	*CC BY-NC*	http://doi.org/10.17632/35sc3vnrtg.2
*D02*	Chamber body	*PDF*	*CC BY-NC*	http://doi.org/10.17632/35sc3vnrtg.2
D03	Chamber lid	*PDF*	*CC BY-NC*	http://doi.org/10.17632/35sc3vnrtg.2
D04	Cylinder mount	*PDF*	*CC BY-NC*	http://doi.org/10.17632/35sc3vnrtg.2
D05	Lid to body mount	*PDF*	*CC BY-NC*	http://doi.org/10.17632/35sc3vnrtg.2
D06	Control box, bottom level	*PDF*	*CC BY-NC*	http://doi.org/10.17632/35sc3vnrtg.2
D07	Control box, top level	*PDF*	*CC BY-NC*	http://doi.org/10.17632/35sc3vnrtg.2
D08	Control box, external view	*PDF*	*CC BY-NC*	http://doi.org/10.17632/35sc3vnrtg.2
D09	Control box, electrical scheme	*PDF*	*CC BY-NC*	http://doi.org/10.17632/35sc3vnrtg.2
D10	Control box, drill holes	*PDF*	*CC BY-NC*	http://doi.org/10.17632/35sc3vnrtg.2
D11	Holes in chamber walls	*PDF*	*CC BY-NC*	http://doi.org/10.17632/35sc3vnrtg.2
D12	Control box, fan and vent	*PDF*	*CC BY-NC*	http://doi.org/10.17632/35sc3vnrtg.2
BOM	Bill of materials	*Excel*	*CC BY-NC*	http://doi.org/10.17632/35sc3vnrtg.2
BI	Build instructions	*PDF*	*CC BY-NC*	http://doi.org/10.17632/35sc3vnrtg.2
CAD	Chamber CAD model	*CAD*	*CC BY-NC*	https://doi.org/10.17632/35sc3vnrtg.2
CADa	Chamber CAD animation	*Video*	*CC BY-NC*	https://doi.org/10.17632/35sc3vnrtg.2

Design sheets D01-D12 show photos and schematics of different parts of the construction, where for each component the designator, source and required quantity are shown. Bill of materials is an Excel sheet with characteristics and costs for every component (the same designator codes used). Build instructions show the building process with photos and schemes. CAD model (FreeCAD FCStd file) shows the main details in the chamber construction and connection between them in 3D. CAD animation is a video file, recorded from the CAD model I action – view of the chamber from different angles, and opening-closing of the chamber.

## 4 Bill of materials summary

The bill of materials (http://doi.org/10.17632/35sc3vnrtg.2) is compiled for a single chamber. Many basic components are widely available and may be sourced from alternative suppliers at slightly varying prices; here, we provide one example source per item as a reference. Detailed specifications and component images are included in the design files D01–D12.

All prices are converted to Euros, excluding VAT for the suppliers offering invoice payments. Costs are rounded to the nearest whole number. Small components often come in bulk; listed prices reflect the estimated cost per unit. Shipping costs are not included.

## 5 Build instructions

Detailed, step-by-step assembly instructions with illustrations for each step are available at repository http://doi.org/10.17632/35sc3vnrtg.2.

Building automatic modular chambers involve assembling chamber bodies and lids, setting up and assembling control boxes, and assembling all parts together. Assembly of control boxes is more convenient in a stationary environment (a laboratory or workshop); assembly of chambers themselves can be done in the field, which is particularly useful for remote study sites. Based on our field experience, a single chamber can be assembled in approximately one hour by one person using a small toolset, provided the control box is preassembled.

To ensure proper alignment of components, a flat, hard surface (e.g., a table or sturdy box) is helpful during assembly. The construction process requires only basic hand tools, such as wrenches, screwdrivers, hand drill, etc. Standard safety precautions when working with hand tools and pneumatic components should be taken.

### 5.1 Chamber body assembly

• Prepare eight 580 mm profiles **1.01** and four 500 mm aluminum profiles **1.02**.
• Fit the slot cover **1.06**/**1.07** into aluminum profile slot and cut it exactly at profile length.
• Repeat the same for all profiles; 580 mm ones should have one side with slot cover, 500 mm ones – two adjacent sides.
• Mount corner pieces **1.03** on both sides of each 500 mm profile **1.02**.
• Gently knocking with a soft mallet, push polycarbonate side sheets **1.05** into slots of 580 mm profiles **1.01**. The sheet should protrude equally about 5 mm from each side. Prepare four sheets with two aluminum profiles on the longer side of each.
• For two of these sheets, connect 500 mm profiles **1.02** on each short side and push them all the way in, gently knocking with a soft mallet.
• Connect all four sides together.
• Make sure the chamber has 90° angles between all sides. Push all sides fully together with a mallet and fully tighten all screws in the corner pieces.

The chamber body is ready.

### 5.2 Chamber lid assembly

• Screw fittings **2.06** on two pneumatic cylinders **2.05**.
• Fix two cylinder mounts **2.03** on each of two pneumatic cylinders 2.05 with 22 mm nuts **2.07** (one nut comes with each cylinder, the second should be purchased additionally). The cylinders should be mounted mirror-symmetrically to each other, so tube fittings look to the same side.
• Mount the two modules produced at step 3 to two 1500 mm beams **2.01** according to the following drawing. Make sure all distances are exact, and all angles are right. Fix the construction with screws **2.15** and t-nuts **2.16**.
• Take two polycarbonate sheets **2.02** and drill one 5 mm hole in each, exactly in the middle of the long side, 20 mm from the edge.
• Put polycarbonate sheets **2.02** inside the inner slot of the lid construction (beams **2.01**) so they can slide to the center and back. Mount the connection between the sheets and the piston of pneumatic cylinders **2.05** in the following order (from below upwards):
a) Screw **2.10**
b) Washer **2.13**
c) Polycarbonate sheet **2.02**
d) Washer **2.13**
e) Nut **2.11** – tighten to a)
f) Thin nut **2.12**
g) Nut **2.11** (three nuts in a row give the necessary distance between the piston and the lid)
h) Angle **2.09**
i) Nut **2.11** – tighten to g)
• Fix the pistons of the pneumatic cylinders **2.05** to the upper hole of the angles **2.09** with two nuts **2.08** (one nut comes with each cylinder, the second should be purchased additionally).
• Close the lid, moving two sheets **2.02** to the center. If necessary, adjust the nuts to achieve tight contact between the sheets in closed state.

The chamber lid is ready.

### 5.3 WiFi relay board setup

The WiFi relay board **3.02** should be configured to work in a wireless network at the site where the chambers will be deployed. The configuration process is described in board online manual [21].

First, connect the board to a PC by USB cable, and power the board by external 12 V DC. Then configure WiFi settings (SSID, security type, password) according to your WiFi environment. It could be recommended to keep automatic (DHCP) IP settings for all the boards at this stage; later specific IP addresses for each board can be either set via DHCP server configuration, or by board configuration via WiFi connection. However, changing SSID, security type and password to WiFi network is possible only via USB connection. Thus, it should be done before mounting the boards inside control boxes.

An alternative approach is to set up a WiFi network, corresponding to the default WiFi002 board settings: SSID = “Workshop”, security = WPA2, password = “password”. Then brand-new boards will connect to this network without any prior configuration.

After the board is connected to WiFi, it can be accessed by its IP address. If the board has HTTP authentication activated, try default username = “admin” and password = “password”. More settings can be adjusted, firmware updated, etc.

Testing of the board relay functions can be done via its web interface (open a browser and type in the IP address of the board). The simplest direct commands can be sent to the board via HTML. If, for example, the board IP address is 192.168.0.99, then the commands look as follows:

http://192.168.0.99/io.cgi?DOA1 – activate relay 1.
http://192.168.0.99/io.cgi?DOA2 – activate relay 2.
http://192.168.0.99/io.cgi?DOI1 – deactivate relay 1.
http://192.168.0.99/io.cgi?DOI2 – deactivate relay 2.

More sophisticated types of communication with the board are described in its online manual [21].

### 5.4 Control box assembly

• Solder terminal block **3.05** to voltage regulator **3.04** (VIN, GND and VOUT holes). Shorten the power plug **3.06** wires to about a size of the voltage regulator (13 mm).
• Connect the wires from the power plug to the terminal block, so that plus (wire marked by white color, connected to the inward pole of the plug) is connected to VOUT and minus (wire connected to the outward pole of the plug) is connected to GND. Connect a blue wire to GND (together with the ground wire from the power plug), and a red wire – to VIN.
• Connect terminal blocks **3.11**, **3.12 and 3.13** by two protection diodes **3.14** as shown below. Fold the construction to be more compact.
• Connect three push-in fittings **2.06** to the 5/2 solenoid valve **3.07** in positions 1, 2 and 4. Positions 3 and 5 can be left empty.
• Prepare the box 3**.01**: drill holes according to the design sheet D10, then fit two grommets **3.19** into 9 mm holes on the front and back walls, and three grommets **3.20** into 5.5 mm holes on the top and bottom walls.
• Cut about 40 cm of the fan power cable **3.10** (female connector end), strip the ends of the wires.
• Mount all components of the bottom level of the control box according to the design sheet D06 and wiring scheme D09. The relay board **3.02** is fixed to the bottom of the box by two screws **3.09** (the holes have slightly different distances, but still possible to adjust). The voltage regulator **3.04** does not require any fixation, as it holds on to the relay board **3.02** by the power plug **3.06**. The valve **3.03** is holding on the tube adaptors **3.18**, tighten in the holes of the front and back walls of the enclosure **3.01**. Terminal blocks **3.11**, **3.12** and **3.13**, connected by diodes **3.14** fit exactly in the space between the valve **3.03** and the enclosure wall.
• Mount the 5/2 solenoid valve **3.07** on the top level of the control box, according to the design sheet D07. The valve is hanging few millimeters above the relays of **3.02**, being held by two elbow adapters **3.21** tighten in the holes of the top wall of the enclosure **3.01**, and the connector **3.23** via adapter **3.22** holding on the bottom wall.
• Close the lid of the enclosure **3.01**, so the ready control box looks as shown at the design sheet D08.

### 5.5 Assembling all together

• Assemble the fan module – connect the fan **3.28** to the fan mount **3.30** using the screw **3.31**. Connect wires from the fan to the power plug **3.29**. The polarity is marked on the plug (plus is connected to the inner contact).
• Mount the lid on the chamber body: Place the chamber body on a flat surface and make sure all the angles are right. Put the lid on top of the body, aligning them symmetrically. Slide t-nuts **2.16** into side slots of long beams **2.01**. These t-nuts can be slide only from the end of the beams; two should go to the back side of the chamber, four – to the front side (two of them will be needed to mount the control box). For chamber body use t-nuts **2.17** (see the design sheet D05) – they can be injected in the middle of the beam, when the ends are blocked. Use joining plates **2.14** and screws **2.15** to fix connection at four corners.
• Drill holes according to the design sheet D11:
• In the front wall of the chamber – two holes, 9 and 12 mm diameter;
• In the rear wall of the chamber – one hole, 9 mm diameter.
• It’s recommended to drill these holes after the chamber body is assembled. The size of polycarbonate sheets **1.05** is slightly smaller than the maximal size that can fit in the aluminum frame slots, to allow thermal expansion in hot weather. Thus, if the holes are positioned relative to the edges of polycarbonate sheets, they would be unprecise relative to aluminum frame during assembling. The distances shown in the design sheet D11 are relative to the aluminum frame.
• Fit the rubber grommet **3.26** into 12 mm hole on the front side, and two rubber grommets **3.19** into 9 mm holes on the front and back sides of the chamber.
• Mount the control box on the front wall of the chamber: fit 6 mm adaptor from the back side of the control box into the hole in the front wall of the chamber; fix the control box with two screws **3.27** connected through the upper holes of the box 3.01 to t-nuts **2.60** slid into the lid beam **2.01**.
• Fit the end of the fan power cable **3.10** into the hole near the control box. Attach the fan **3.28** by the mount **3.30** to the lower beam **1.01** inside the chamber, above the gas inlet – plug the fan vertically and turn 45 degrees to fix. Connect the fan power plug **3.29** to the power cable **3.10**.
• Connect the pneumatic cylinders **2.05** to the control box. First, connect straight adaptors **3.22** and Y adaptors **3.24** to the elbow adaptors **3.21** on the top of the control box.
• Connect Y adaptors to pneumatic cylinders by 4 mm tubing **3.32**. Rightmost adaptor should be connected to inner inlets of both cylinders (pressure applied to open chambers when the valve **3.07** is unpowered); leftmost adaptor – to outer inlets (pressure applied to close chambers when the valve **3.07** is powered).
• Prepare PVC seals **2.04** – cut two 578 mm pieces (just a bit shorter than horizontal beams **1.01**). Try to keep the right angle of the cut. Close the chamber, make sure two polycarbonate sheets **2.02** meet exactly in the middle of the chamber. Stick the PVC seals to the outer parts of sheets **2.02**, so they touch the beams **1.01**.

The chamber is ready. Next step is to install it in the field and connect power and gas tubing.

## 6 Operation instructions

Once assembled and installed, each chamber requires the following to operate:

• Power supply, 24 V DC
• WiFi coverage, 2.4 GHz
• Compressed air, 2–3 bar
• Sample air tubing, connected to a greenhouse gas analyzer

The valves tolerate ± 10% voltage fluctuation, while the voltage regulator and the fan can handle even greater deviations. Since the main force for chamber operations is pneumatic, power consumption is low:

• Idle chamber: 1.1 W
• Active open chamber: 5.9 W
• Active closed chamber: 7.2 W

Power supply for each chamber, or each group of adjacent chambers, can be autonomous (e.g., via solar panel and battery). Another approach is to connect power to all the chambers from the same central source. Individual chambers can be connected in series along a shared power cable. Even with long cables (e.g., 300 m at our Norwegian site), voltage drop remains within acceptable limits.

Compressed air can be distributed similarly, as long as each chamber receives 2–3 bar at the pneumatic inlet. At our sites, power, compressed air, and sample air tubing are routed together and split near each chamber. (Fig. 3).

In a typical multichamber setup one chamber is always active (sample air valve open, air is directed to the gas analyzer), all other chambers are idle (sample gas valves closed, lids open). The active chamber can be closed (during the measurements) or open (ventilating few minutes before and after the measurements). The fan operates when the chamber is active (wired in parallel with the sample air valve, see D09). It mixes the air inside the closed chamber to ensure equal concentrations of gases in the whole headspace, and helps to ventilate the open chamber to ensure ambient concentrations in the headspace. Measurement and ventilation timing should consider:

• expected flux rates – how fast the concentration in the chamber expected to change
• gas analyzer sensitivity, frequency of concentration measurements and air displacement time
• tubing length – how fast air from the chambers reaches the analyzer
• practical scheduling (e.g., fitting full cycles into 24-hour periods)

The measurement time (closed chamber) can be from 2 to 3 min (CO_2_ exchange in a productive ecosystem, CH_4_ emission in wetland) to 15 min or more (N_2_O fluxes, CH_4_ fixation). The ventilation time should be at least two minutes before and after the measurement, plus lag time depending on tubing length. The time needed for each measurement is the only practical limitation for the number of chambers in a setup – more chambers mean longer cycles and lower time resolution of measurements from each individual chamber.

Chambers are controlled via WiFi from any networked device (e.g., computer, datalogger, Arduino). Each chamber should have a unique IP address (static or DHCP-reserved). Commands sent to this address activate/deactivate the chamber, and open/close it. The WiFi relay board 3.02 (see D06) supports different communication protocols, described in its online manual [21]. The simplest direct commands can be sent to the board via HTML. E.g., for the board with IP address 192.168.0.99 the commands are:

• https://192.168.0.99/io.cgi?DOA1 – activate relay 1 (activate the chamber)
• https://192.168.0.99/io.cgi?DOA2 – activate relay 2 (close the lid)
• https://192.168.0.99/io.cgi?DOI2 – deactivate relay 2 (open the lid)
• https://192.168.0.99/io.cgi?DOI1 – deactivate relay 1 (deactivate the chamber)

The board responds with an acknowledgement when the command is executed. More advanced TCP/IP communication protocols allow to establish contact with each board to make sure each chamber is online even while it’s idle. If a chamber falls offline, the program can skip it and continue with other chambers.

For the simplicity of the construction, chambers do not include physical sensors to confirm lid position. However, the validity of operations can be confirmed indirectly by:

• relay board responses
• changes of pressure in the pneumatic line
• sample gas concentration patterns

**Fig. 8:**
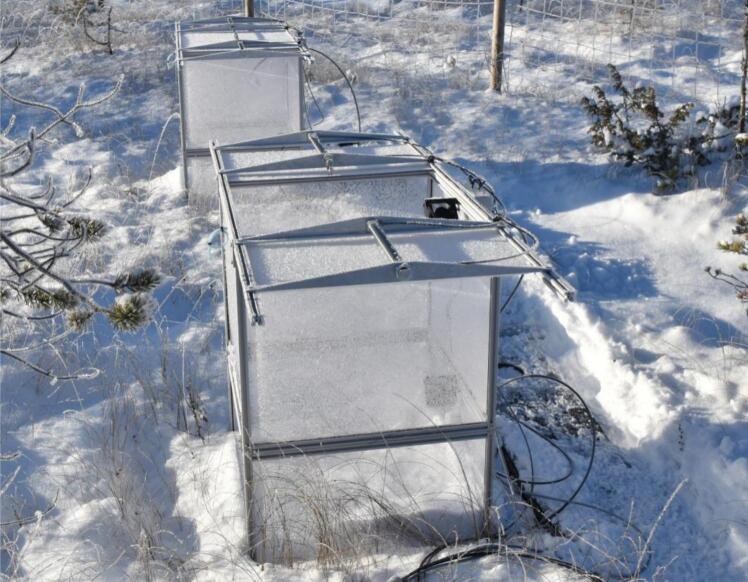
Double-high chambers are ready for deep snow cover (Puukkosuo).

Depending on individual site characteristics, chambers can be installed directly onto the ground (flat wetland surface with high standing water), or on frames (uneven terrain, soil with high air porosity). Frames can be built from the same aluminum profiles and polycarbonate sheets. Similar construction can be used to extend chamber height for measurements on tall vegetation or deep snow cover in winter (Fig. 8).

If frames are installed in many plots, a limited number of chambers can be rotated between plots to increase spatial coverage. As long as all plots are within WiFi range, chambers operate the same way regardless of their positions.

In general, during snow-free conditions chambers work autonomously and do not need any service. In winter, icing can prevent lid closure; then manual ice removal is needed. Increasing the pressure in the pneumatic line (to about 3 bars instead of normal 2 bars) helps to ensure reliability at negative temperatures. To avoid freezing of fans, they can be rewired inside the control box to be powered all the time.

If some construction elements break down during the installation or operations, they can be easily replaced. Polycarbonate sheets become less transparent after few years of UV exposure; replacing the lid sheets is very easy and can be done in few minutes, replacing the wall sheets takes more effort but still doable in field conditions. Replacing the pneumatic cylinders is recommended when they become leaky (after 2–3 years of operation, especially in winter conditions). This can be also done on site and takes few minutes.

### 6.1 Safety and reliability

During normal operations, the chambers can be considered safe. For the risk assessment in abnormal situations, two potential aspects should be considered: pneumatic and electrical risks.

Pneumatic system is designed for pressures in the range of 1..2 bars in snow-free conditions, and up to 3 bars in harsh winter (snow, icing) conditions. These pressures are far below the maximum operating pressure for pneumatic cylinders **2.05** (10 bar), solenoid valves **3.07** (7 bar), tubing (50 bar) and fittings (14–20 bar). However, common ways of compressed air supply in the field are compressors and gas cylinders, where primary pressure is higher (6 – 10 bar for compressors, 50 bars and higher for cylinders). Risk assessment and protective measures (e.g. pressure relief valve) should be performed for each specific source of compressed air.

The described automatic chamber construction does not involve any anti-pinch mechanism, as it was considered not necessary under the normal operating conditions, and could be expensive and complicated to implement. For example, EU and US standards for power windows in modern automobiles [22] state the maximum force a power window is allowed to exert on any object is 100 N; the theoretical (without friction and pressure loss) pneumatic cylinders **2.05** force when closing the chamber is about 94 N at 3 bars. Thus, in terms of pinch, the chambers are as safe as modern car windows, if the operational pressure is kept within the specifications.

Regarding power supply, the chambers require 24 V DC voltage with toleration of ±10% voltage fluctuation. The described construction does not involve any protection from reverse polarity or overvoltage; such protection, as well as its risk assessment, must be resolved individually at each specific site. In our experience, reverse polarity causes burning of protection diodes **3.14**, fan **3.28** and voltage regulator **3.04**. Voltages above 24 V are not utilized in any part of the described setup, which is far below the threshold of 50 V which is generally considered potentially dangerous. However, if the whole site operations involve higher voltages (e.g. gas analyzer, computer, air compressor), safety measures (e.g. fusing, grounding) and risk assessment should be performed accordingly.

**Fig. 9:**
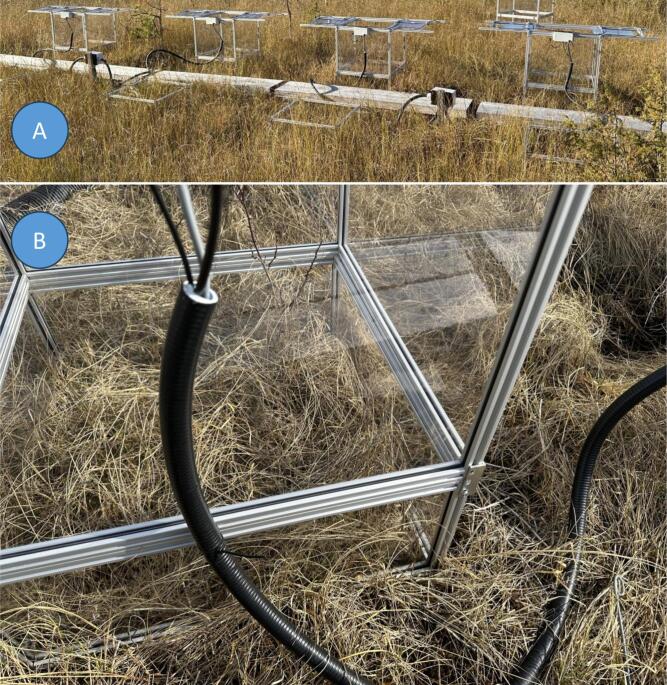
Each chamber is weekly moved from one frame to the other (Puukkosuo). A: Chambers on their frames at one side of the boardwalk, alternative frames on the other side. B: close-up of the chamber, standing on the frame; cable and tubing in the conduit fixed to the chamber edge.

Strain relief can be considered for power cables and tubing. The described construction involves the connector **3.15** for the power cable, connectors **3.23** for pneumatic tubing and **3.25** + **3.18** for sample gas tubing, which are enough if the chamber stays in the same plot. If moving the chambers without disconnecting cables and tubing is planned, additional strain relief can be achieved by fixing cables and tubing (optionally in a conduit) to a chamber edge (Fig. 9B).

For an autonomous measurement system it’s important to account for power loss situations. If our chambers lose power, the sample gas valve **3.03** closes (so air from this chamber does not go to the analyzer and does not interfere with other chambers), the compressed air valve **3.07** switches to the default state (see Fig. 3), and the lid opens – if it was closed – by the force of compressed air in the pneumatic system. Even if the whole system, including the air compressor, loses power, the residual pressure in the compressor tank is enough to open the chambers which were closed. Thus, the artificial disturbance for the ecosystem is minimized.

### 6.2 Technical specifications

Main technical specifications for automatic chambers are shown in Table 1. Chamber internal volume is given for a chamber standing on a rigid flat surface, the real volume of every chamber should be measured in the field after installation. Compressed air consumption implies no leakage.

**Table 1:** Main technical specifications for one chamber, “standard” size.

External height	550 mm
External length (incl. lid beams)	1500 mm
External width	620 mm
Effective covered area	600 × 600 mm, 0.36 m^2^
Internal volume	600 × 600 × 500 mm, 0.18 m^3^
Weight	15 kg
Operating temperature, pneumatics	−20 … +80 °C
Operating temperature, electronics	−20 … +70 °C
Operating temperature, field validated	−30 … +30 °C
Operating humidity, field validated	Up to 100%, rain and snow tolerate
Power supply	24 V DC, ±10%
Power consumption, idle	1.1 W
Power consumption, active, open	5.9 W
Power consumption, active, closed	7.2 W
Compressed air supply pressure	1 … 2 bar
Compressed air supply pressure, snow/ice conditions	2 … 3 bar
Compressed air consumption, per cycle	0.6 l at 2 bar, 0.9 l at 3 bar
Pneumatic tubing connection	4 mm o.d., push-in connector
Sample gas tubing connection	6 mm o.d., push-in connector
Sample gas flow rate	0.1–5 lpm

## 7 Validation and characterization

Automatic chamber systems based on the described modular design – with minor site-specific adaptations – have been successfully deployed over the past years in Arctic and subarctic field studies. Below are our four key validation sites:

### 7.1 Zackenberg I

Arctic fen site in Zackenberg valley, Northeast Greenland National Park (74°29′ N, 20°33′ W), close to Zackenberg Research Station [23]. This site has a twenty-year history of CO_2_ and CH_4_ flux monitoring [9], [20] under Greenland Ecosystem Monitoring Programme (GEM) [24]. New modular chambers came as a replacement for the old chambers, at the existing site infrastructure. During one growing season both old and new chambers were running in parallel to ensure data compatibility. In this site we learned that existing monitoring sites can be easily upgraded with the new generation of automatic chambers within a limited budget. New chambers have proven high reliability and low maintenance demands.

### 7.2 Zackenberg II

Another fen site in Zackenberg valley (74°28′ N, 20°31′ W), established under Greenland Integrated Observing System (GIOS) [25]. At this site modular chambers were installed as a part of a mobile observatory powered by sun and wind [26]. Key requirements were fully autonomous operations and low power consumption. During the first test season the chamber setup worked unattended for 25 days, until it was broken by polar bears. In the second season the unattended period reached 67 days, and again the chambers were broken by polar bears.

We cannot imagine a chamber construction that would be capable of withstanding the destructive power of a polar bear. But we learned how our chambers break in such situations, and how to fix them effectively in field conditions. The corner parts (**1.03**, see D02) break relatively easily, and the construction falls apart without any damage to aluminum frame details and polycarbonate sheets. We had to replace aluminum corners **1.03**, slot covers **1.06** and **1.07**, ripped tubing and power cables. One control box was broken, we replaced it entirely. It took two days for one person on site to fix the setup, that looked completely destroyed.

### 7.3 Puukkosuo

Boreal fen site in the Oulanka National Park in Northeast Finland (66°22′ N, 29°19′ E), close to Oulanka Research Station [27]. Automatic chambers were installed as a part of the EcoClimate long-term, manipulative natural experiment [28]. First two chambers were installed to test the concept of year-round measurements in the area with continental climate and winter air temperatures down to −38 °C. The chambers have been working continuously since October 2021, including four winters. Numerous improvements have been tested, still some maintenance is needed to ensure winter operations. In May 2024 ten more chambers were added to be operated on 20 new plots. These chambers were rotated weekly between two plots during snow-free periods to accommodate reindeer grazing (Fig. 9). During winter these chambers operated stationary.

This project validated chamber performance in harsh winter conditions and led to several design improvements.

### 7.4 Pasvik

**Fig. 10:**
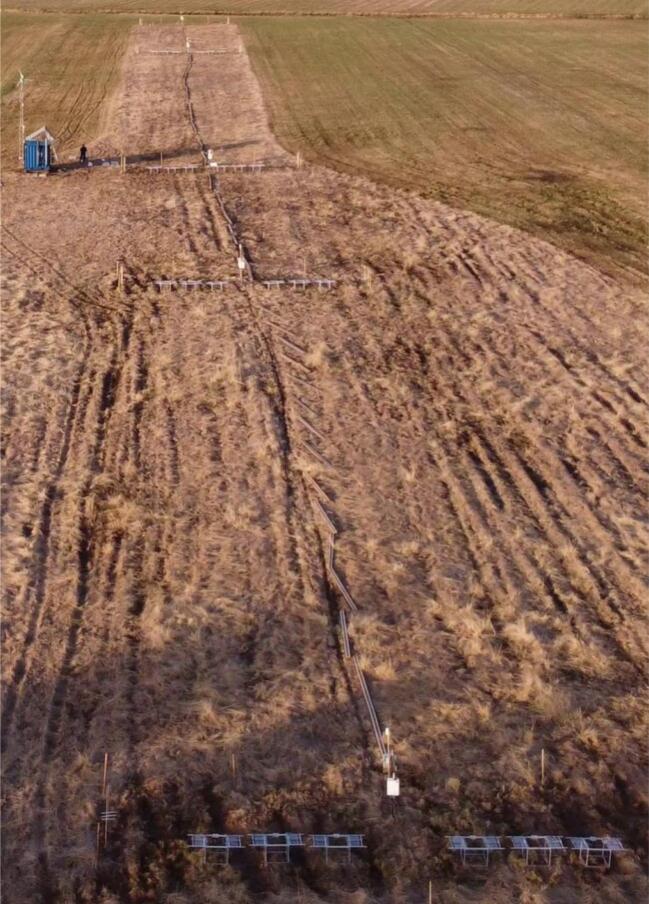
Five plots with six chambers on each, all connected to one analyzer (Pasvik).

Cultivated peatland site in northern Norway (69˚28′ N, 29˚59′ E), close to NIBIO Svanhovd Research Station [29]. There automatic chamber setup was installed as a part of project “Climate smart management practices on Norwegian organic soils” (MYR) [30] and later continued measurements under the project “Monitoring, Reporting and Verification of Soil Organic Carbon and Greenhouse Gas Balance” (MRV4SOC) [31]. At this site a set of 30 automatic chambers operated on 5 plots ∼50 m^2^ each, covering >300 m transect (Fig. 10). For such extended layout wireless operation and chained gas line were essential. We faced challenges of very long tubing and power cables, rodent damage, power issues, etc. Despite these, reliable CO_2_, CH_4_, and N_2_O flux measurements have been achieved with an 8-hour cycle across all 30 chambers during four snow-free seasons (May – November).

### 7.5 Conclusion

Across these four sites, the modular chamber system has demonstrated:

• **Practicality** in diverse field conditions
• **Reliability** over multi-season deployments
• **Affordability** compared to commercial alternatives

With over a decade of cumulative field experience, this setup is recommended for a wide range of GHG flux studies.

## Ethics statements

Not relevant.

## Supplementary materials

Two Supplementary materials SM1 and SM2, presenting applicability of linear model to flux estimation from flow-through chambers (theoretical background and numerical evaluation) can be accessed via the repository https://data.mendeley.com/datasets/rdy8k66z2r/1.

## CRediT authorship contribution statement

**Mikhail Mastepanov:** Writing – review & editing, Writing – original draft, Visualization, Validation, Supervision, Software, Resources, Project administration, Methodology, Investigation, Conceptualization.

## Declaration of competing interest

The author declares that they have no known competing financial interests or personal relationships that could have appeared to influence the work reported in this paper.
