# Supplementary material for: Wirelessly controlled modular automatic chambers for greenhouse gas flux monitoring in natural and agricultural ecosystems
Source: HardwareX. 2026 Apr 30;26:e00782. doi: 10.1016/j.ohx.2026.e00782 (PMC13158385; doi:10.1016/j.ohx.2026.e00782)

D01 General appearance

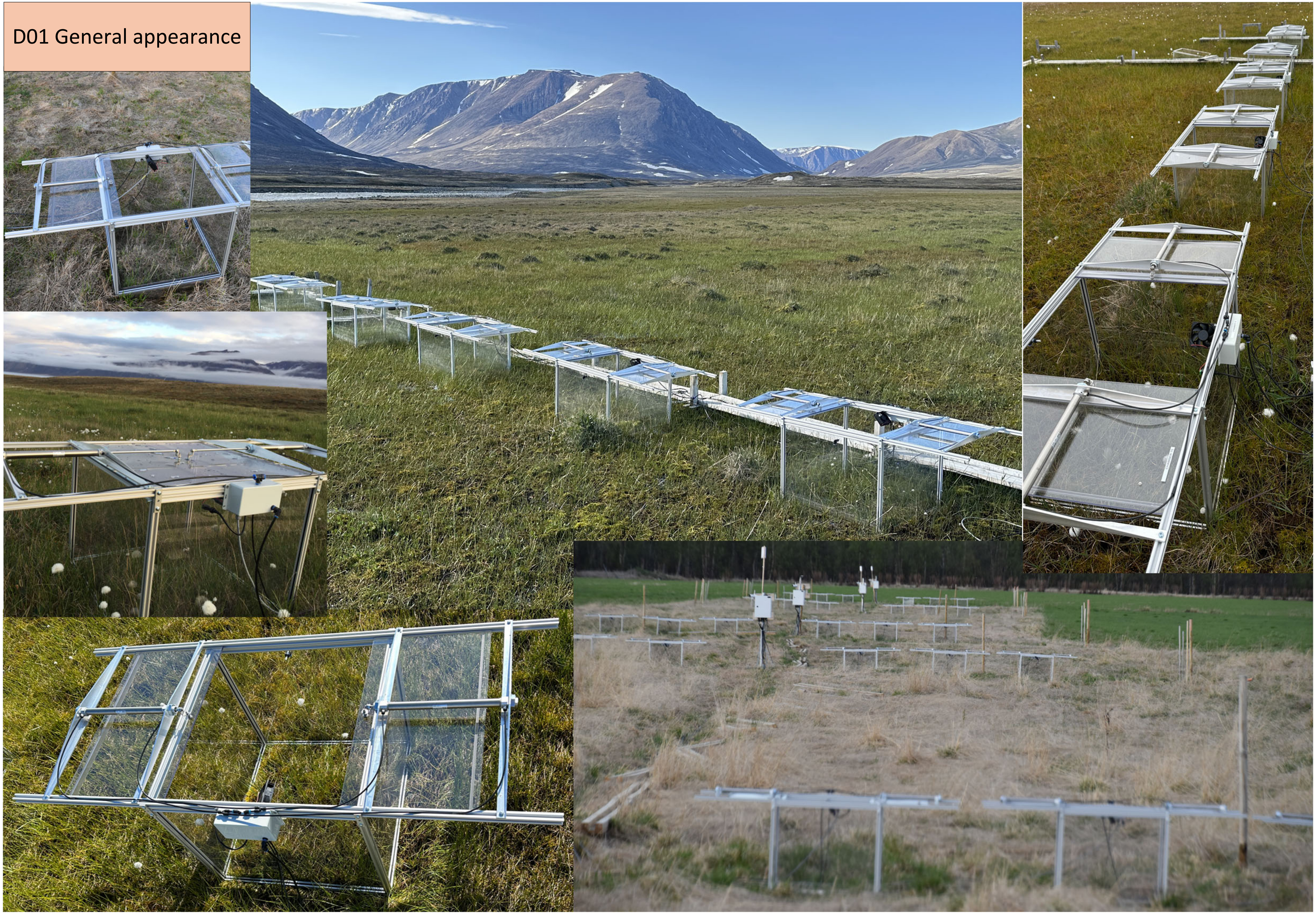

D02 Chamber body

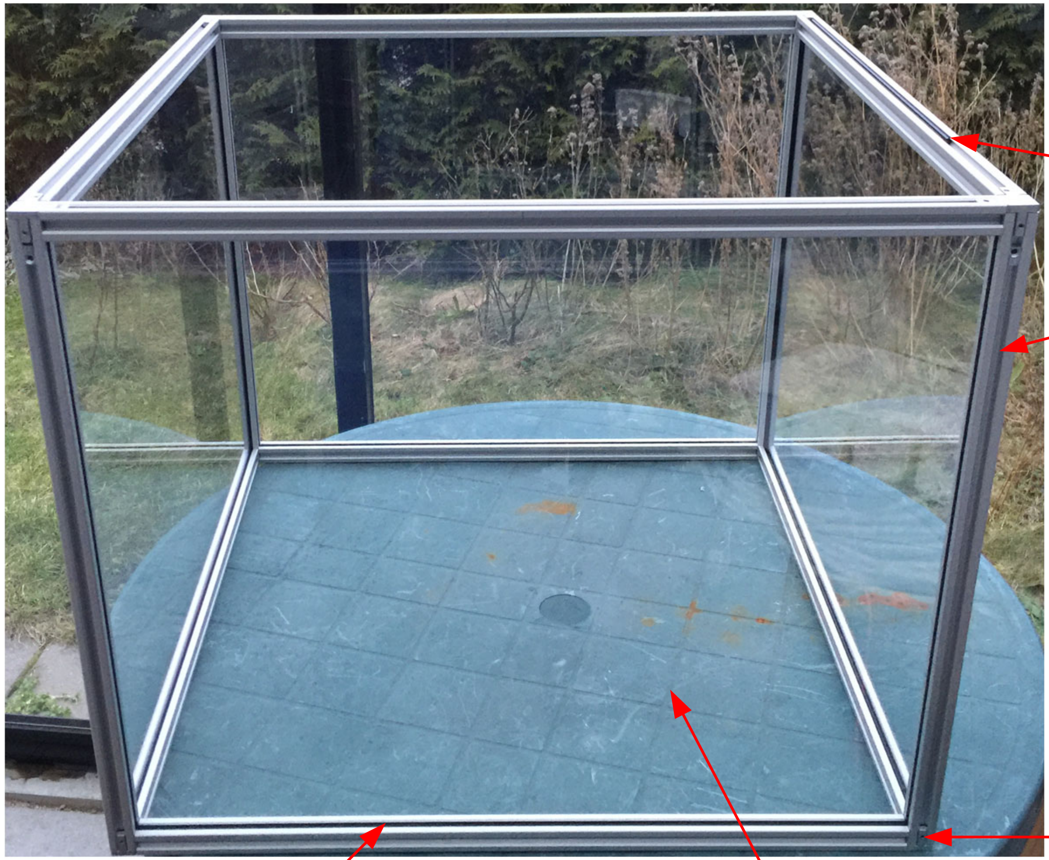

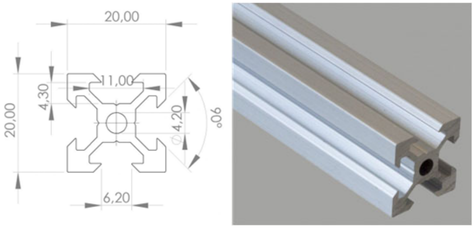

V-SLOT Aluminium profile 20x20 6mm slot

https://www.systeal.com/en/profiles-20-series/1221-v-slot-aluminium-profile-20x20-6mm-slot.html

1.01 (horizontal beam) 580 mm 8 pieces

1.02 (vertical beam) 500 mm 4 pieces

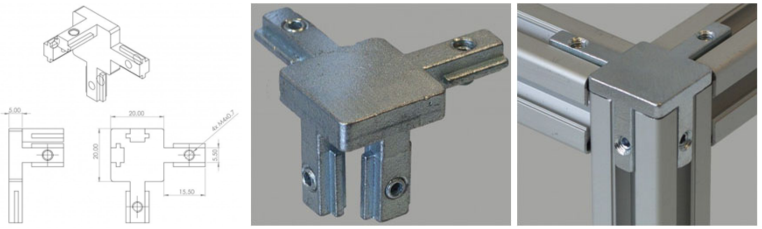

Internal assembly connector - 3 ways for 6 mm slot

https://www.systeal.com/en/assembly-solutions/1180-internal-assembly-connector-3-ways-for-6-mm-slot.html

1.03 8 pieces

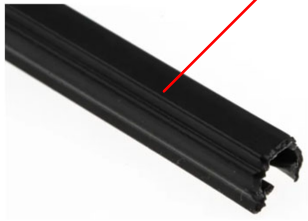

T-Slot Cover 5mm

https://dk.rs-online.com/web/p/daeklister/1809131

1.06 580 mm 8 pieces

1.07 500 mm 4 pieces

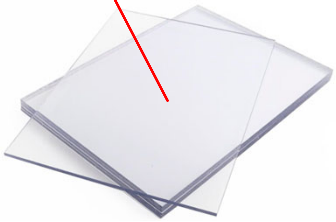

Polycarbonate Transparent 3 mm

https://interglas.dk/shop/polycarbonat-klar-3-749p.html

1.05 590 x 480 mm 4 pieces

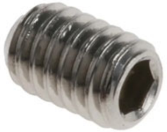

Grub Screw M4 x 6mm

https://dk.rs-online.com/web/p/pinolskruer/0137758

1.04 (spare parts)

D03 Chamber lid

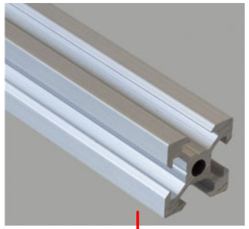

V-SLOT Aluminium profile 20x20 6mm slot

https://www.systeal.com/en/profiles-20-series/1221-v-slot-aluminium-profile-20x20-6mm-slot.html

2.01 (long beam) 1500 mm 2 pieces

Polycarbonate Transparent 6 mm

https://interglas.dk/shop/polycarbonat-klar-6-753p.html

2.02 590 x 330 mm 2 pieces

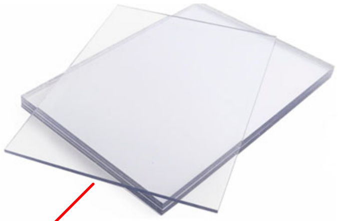

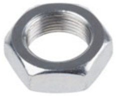

Nut M22

https://dk.rs-online.com/web/p/tilbehor-til-pneumatiske-cylindre-og-akuatorer/1215692

2.07 2 pieces

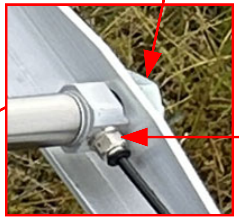

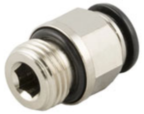

Push-in Fitting 1/8' to 4 mm

https://dk.rs-online.com/web/p/pneumatik-fittings/1761276

2.06 4 pieces

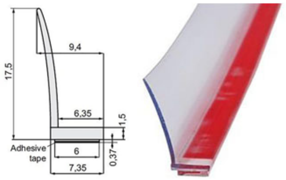

PVC Seal, ADH02

https://interglas.dk/shop/pvc-taetning-fra-1474p.html

2.04 578 mm 2 pieces

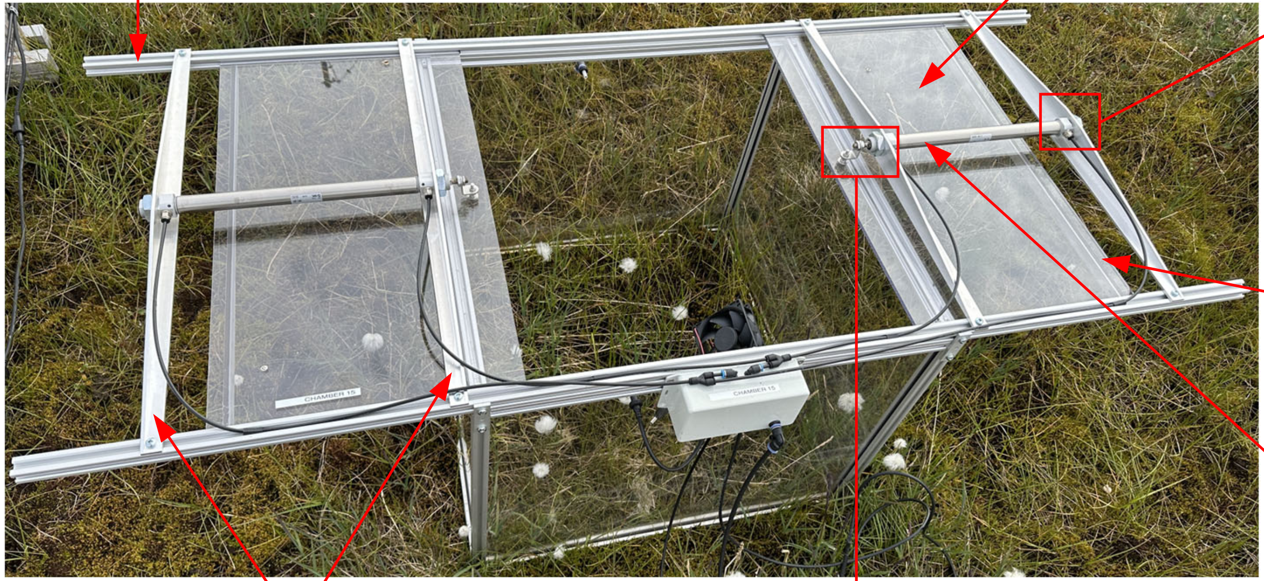

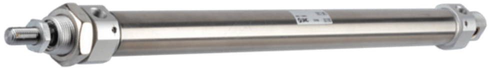

Pneumatic Piston Cylinder, 20mm Bore, 250mm Stroke

https://dk.rs-online.com/web/p/pneumatik-stempelstangscylindre/7006005

Model SMC CD85N20-250C-B

2.05 2 pieces

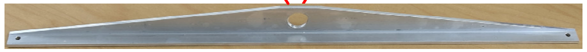

Cylinder Mount

See a separate sheet

2.03 4 pieces

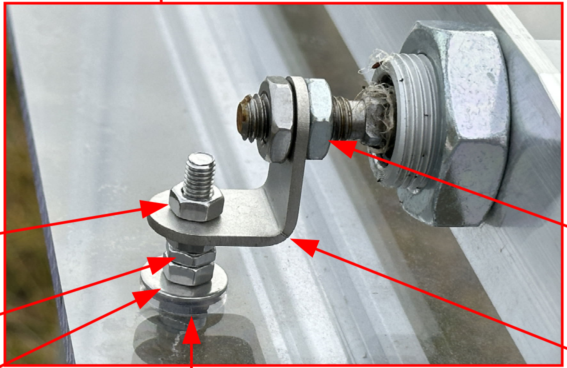

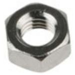

Nut M5, standard

https://dk.rs-online.com/web/p/sekskantmotrikker/0189585

2.11 6 pieces

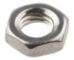

Nut M5, half height

https://dk.rs-online.com/web/p/sekskantmotrikker/1224402

2.12 2 pieces

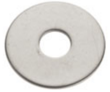

Washer M5

https://dk.rs-online.com/web/p/spaendeskiver/0189642

2.13 4 pieces

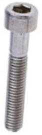

Screw M5 x 30mm

https://dk.rs-online.com/web/p/selvskaerende-gevindformende-skruer/0521535

2.10 2 pieces

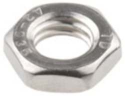

Nut M8 DIN 439B

https://dk.rs-online.com/web/p/sekskantmotrikker/1224404

2.08 2 pieces

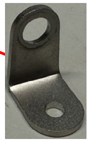

Stainless Steel Angle

https://dk.rs-online.com/web/p/dele-til-montageplads/7866185

2.09 2 pieces

D04 Cylinder mount

2.15 8 pieces
2.16 8 pieces

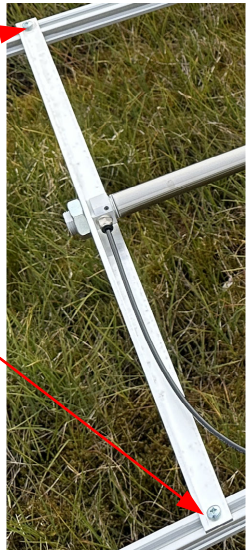

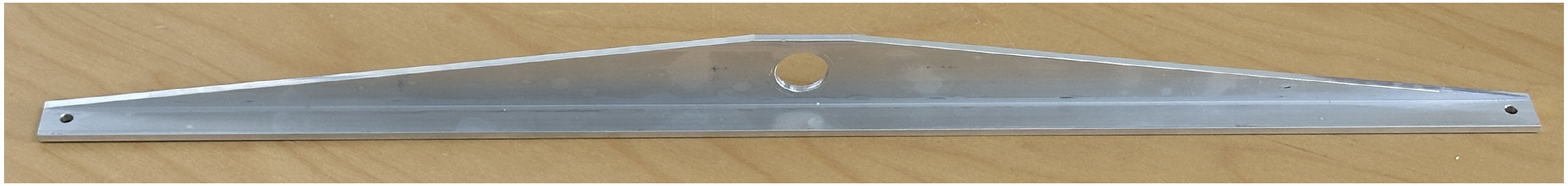

Side view

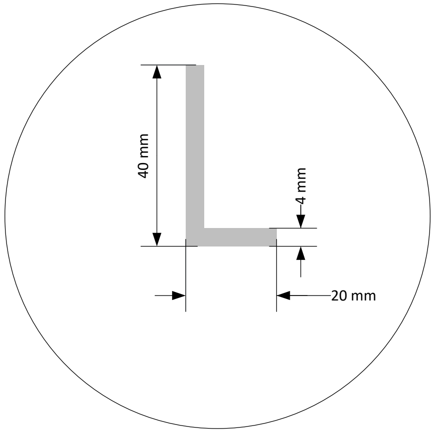

Top view

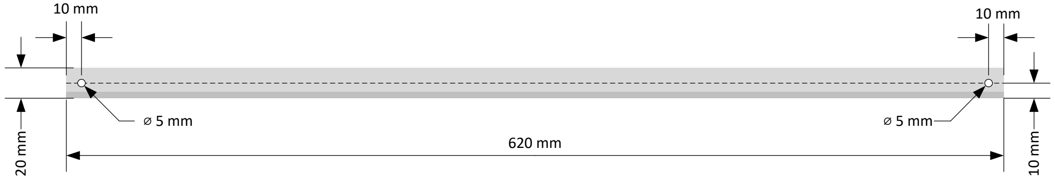

Front view – simple design

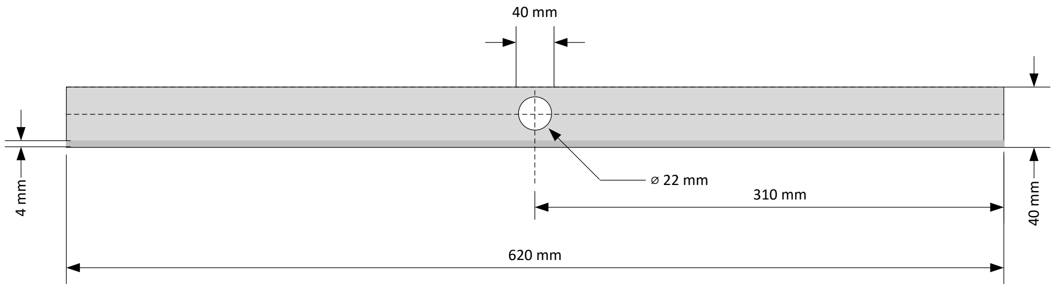

Front view – elegant design

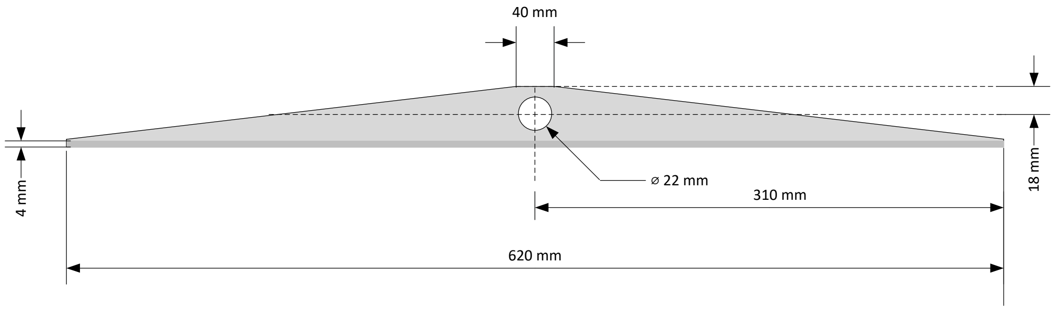

D05 Lid to body mount

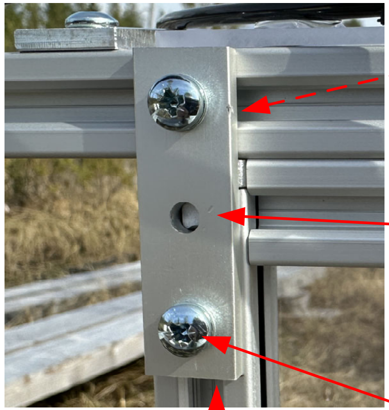

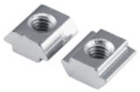

T-nut sliding

https://www.electrokit.com/en/t-nut-square-for-2020-m5-single

2.16 4 pieces

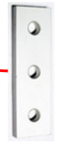

Joining Plate

https://www.electrokit.com/joining-plate-for-2020-3-hole-strip

2.14 4 pieces

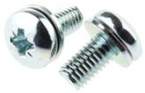

Screw with built-in washer

https://dk.rs-online.com/web/p/skruer-med-skive/0278916

2.15 8 pieces

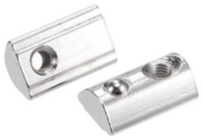

T-Nut spring loaded

https://www.electrokit.com/en/t-nuts-spring-loaded-m5-for-2020-25-pack

2.17 4 pieces

D06 Control box, bottom level

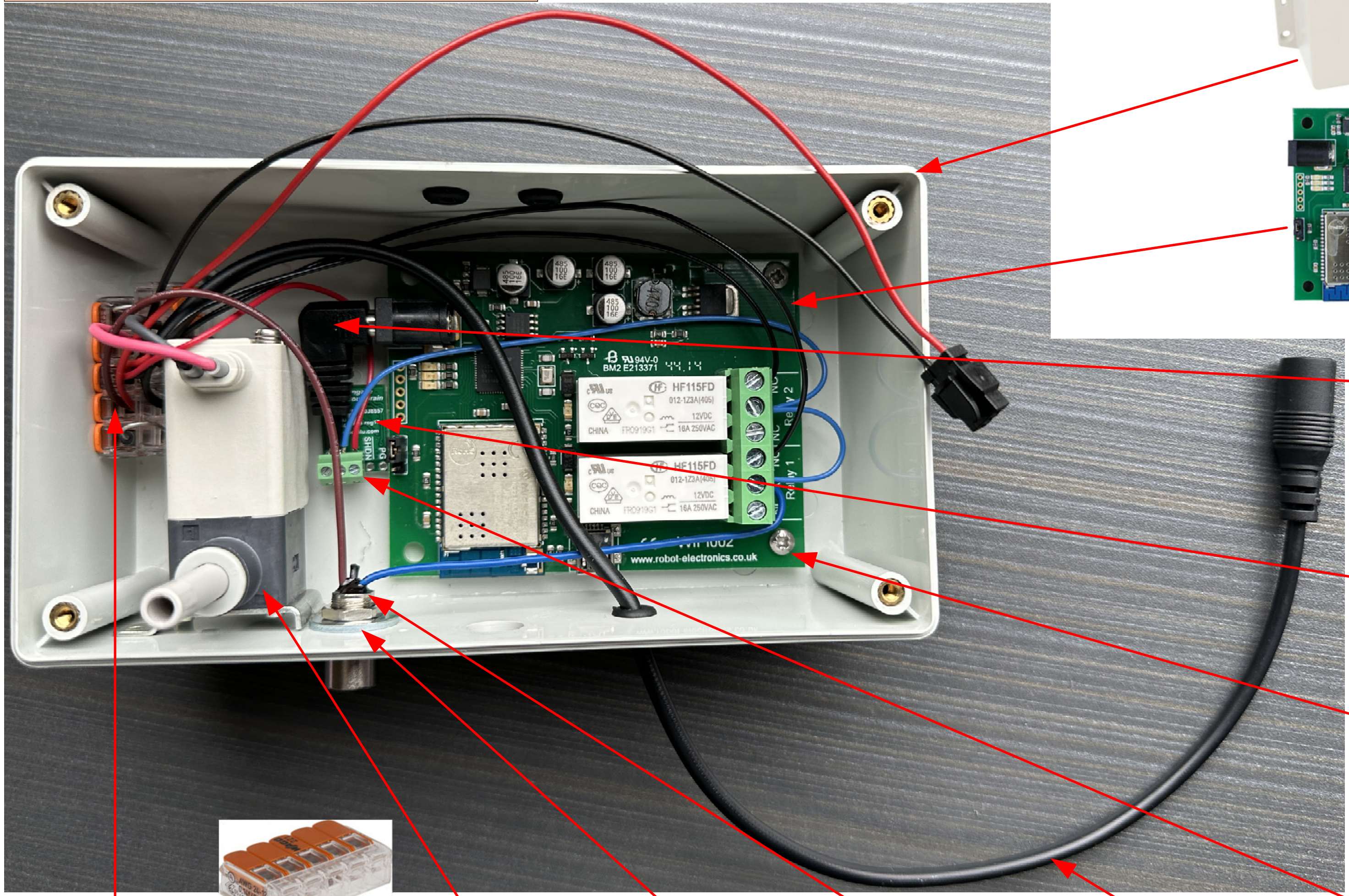

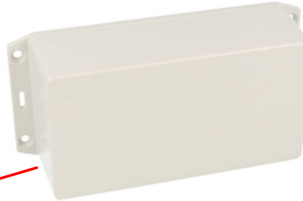

ABS Enclosure
Hammond 1591XXDFLGY
https://dk.rs-online.com/web/p/allround-kabinetter/2287329
3.01 1 piece

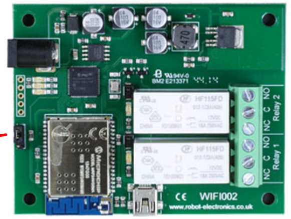

WiFi relay board
Devantech WIFI002
https://www.robot-electronics.co.uk/products/relay-modules/wifi-relay/wifi002-2-x-16a-relay-module.html
3.02 1 piece

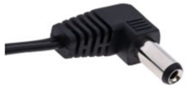

Power Plug
https://dk.rs-online.com/web/p/stromkabler/6563850
3.06 1 piece

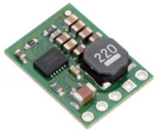

Voltage regulator
https://exp-tech.de/products/pololu-12v-1a-step-down-voltage-regulator-d24v10f12
3.04 1 piece

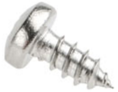

Self Tapping Screw
https://dk.rs-online.com/web/p/selvskaerende-gevindformende-skruer/0521535
3.09 2 pieces

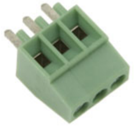

Terminal Block
https://dk.rs-online.com/web/p/printklemraekker/7901092
3.05 1 piece

Terminal blocks
https://dk.rs-online.com/web/p/standardklemmeraekker/8837557
3.11 5-Way 1 piece
https://dk.rs-online.com/web/p/standardklemmeraekker/8837548
3.12 3-Way 1 piece
https://dk.rs-online.com/web/p/standardklemmeraekker/8837544
3.13 2-Way 1 piece

Protection diodes 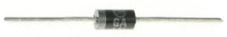
https://dk.rs-online.com/web/p/switching-dioder/7815562
3.14 2-Way 2 pieces

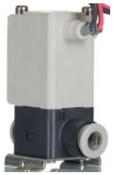

Solenoid Valve
https://dk.rs-online.com/web/p/pneumatik-magnetventiler-pilotstyret/8929993
3.03 1 piece

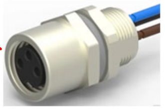

Power connector
https://dk.rs-online.com/web/p/industrielle-cirkulaere-stik/2496334
3.15 1 piece

Washer M8 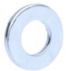
https://dk.rs-online.com/web/p/spaendeskiver/0527634
3.16 1 piece

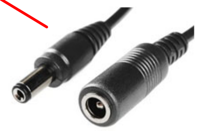

Fan power cable
https://www.wattoo.dk/dc-forlaengerkabel-50-cm-til-led-strips-sort-5v-48v-9903002153
3.10 1 piece

D07 Control box, top level

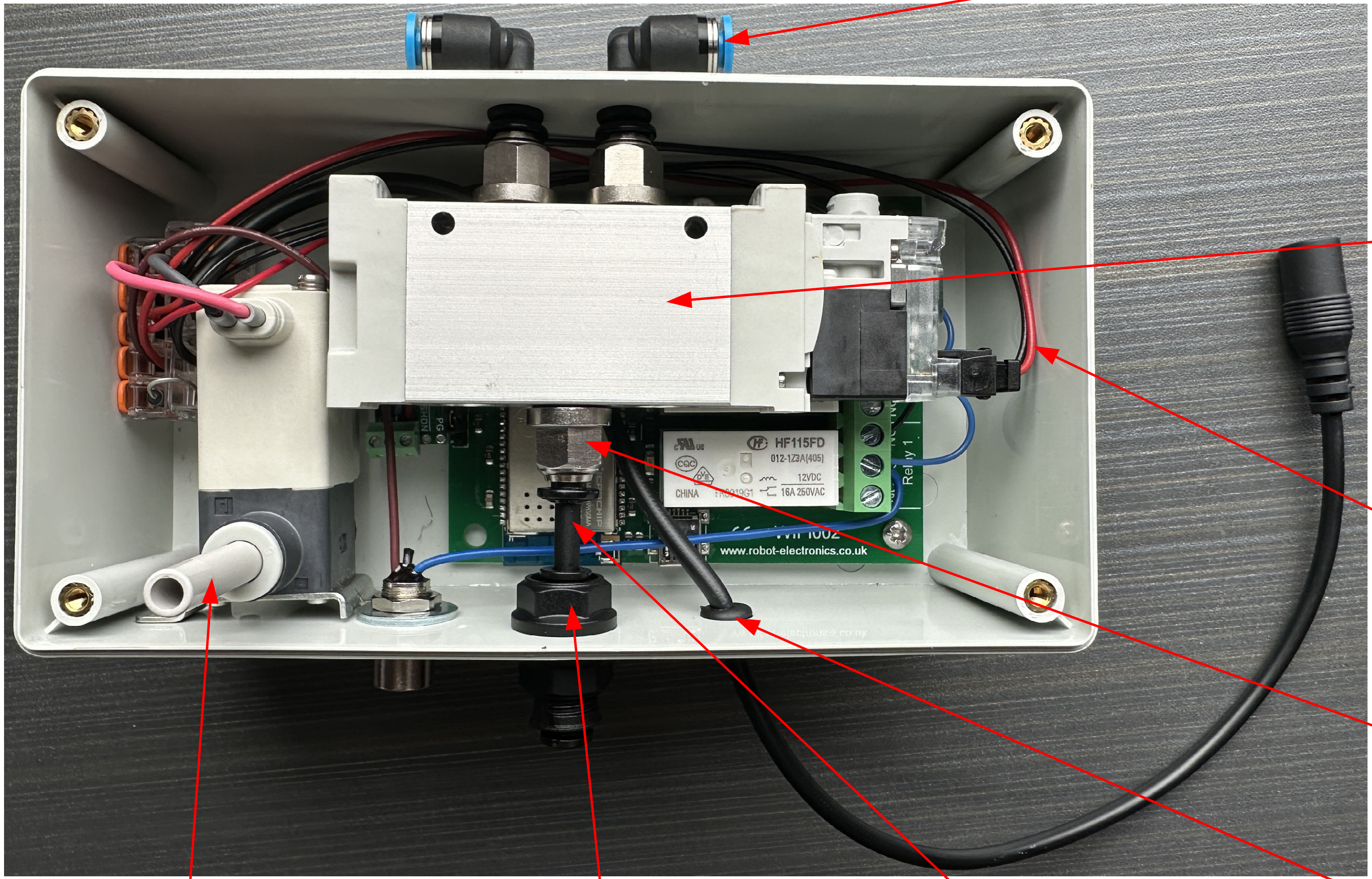

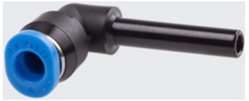

Elbow adaptor 4 mm

https://dk.rs-online.com/web/p/pneumatik-fittings/1216015

3.21 2 pieces

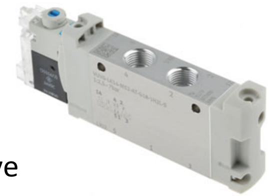

5/2 Solenoid valve

https://dk.rs-online.com/web/p/pneumatik-magnetventiler-pilotstyret/1215889

3.07 1 piece

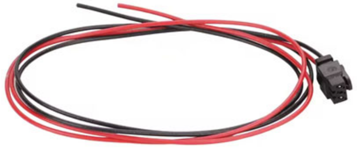

Valve plug with cable

https://dk.rs-online.com/web/p/tilbehor-til-pneumatiske-sensorer-og-switche/1215582

3.08 1 piece

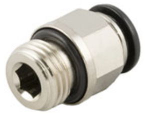

Push-in Fitting 1/8' to 4 mm

https://dk.rs-online.com/web/p/pneumatik-fittings/1761276

2.06 3 pieces

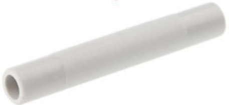

Straight adaptor 6 mm

https://dk.rs-online.com/web/p/pneumatik-fittings/4002909

3.18 2 pieces

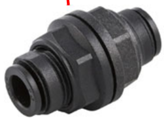

Straight connector 4 mm

https://dk.rs-online.com/web/p/pneumatik-fittings/1761408

3.23 1 piece

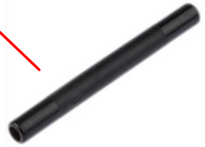

Straight adaptor 4 mm

https://dk.rs-online.com/web/p/pneumatik-fittings/2317057

3.22 1 piece

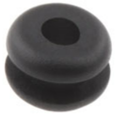

Rubber grommet

https://dk.rs-online.com/web/p/kabelgennemforinger/1366806

3.20 3 pieces

D08 Control box, external view

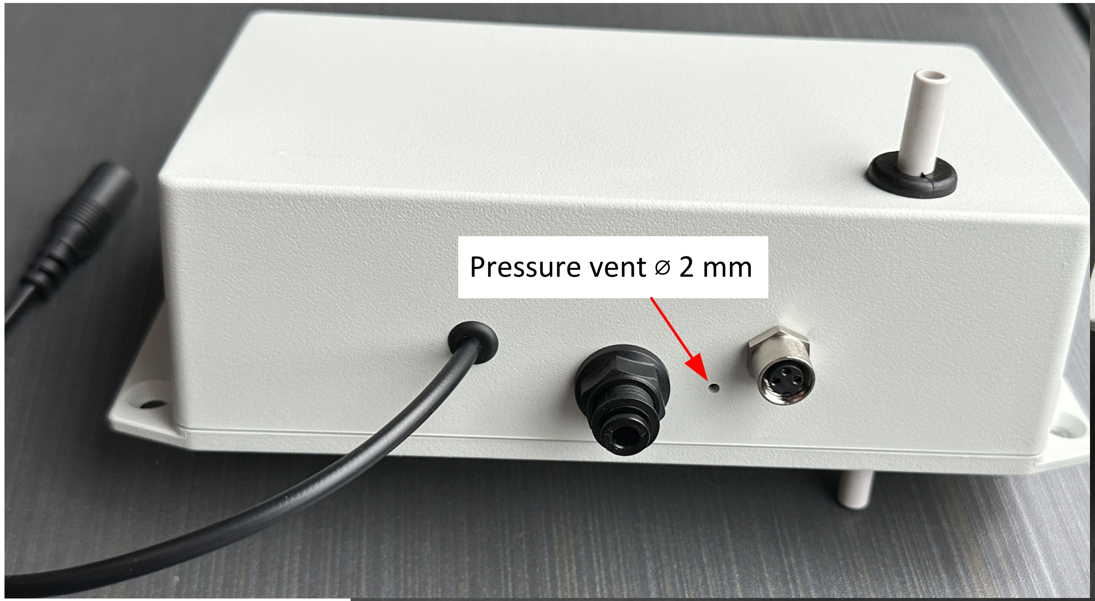

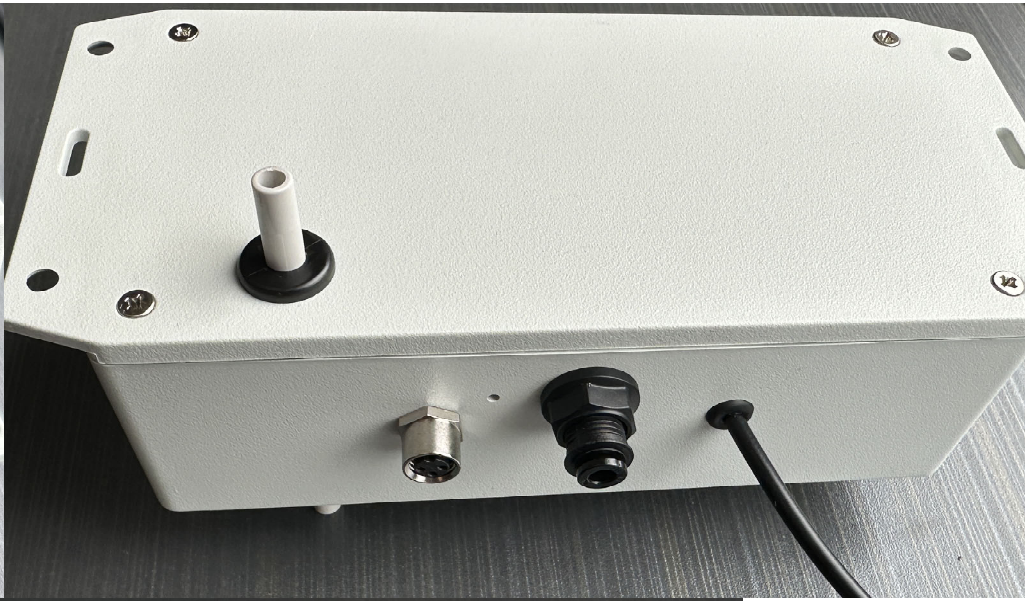

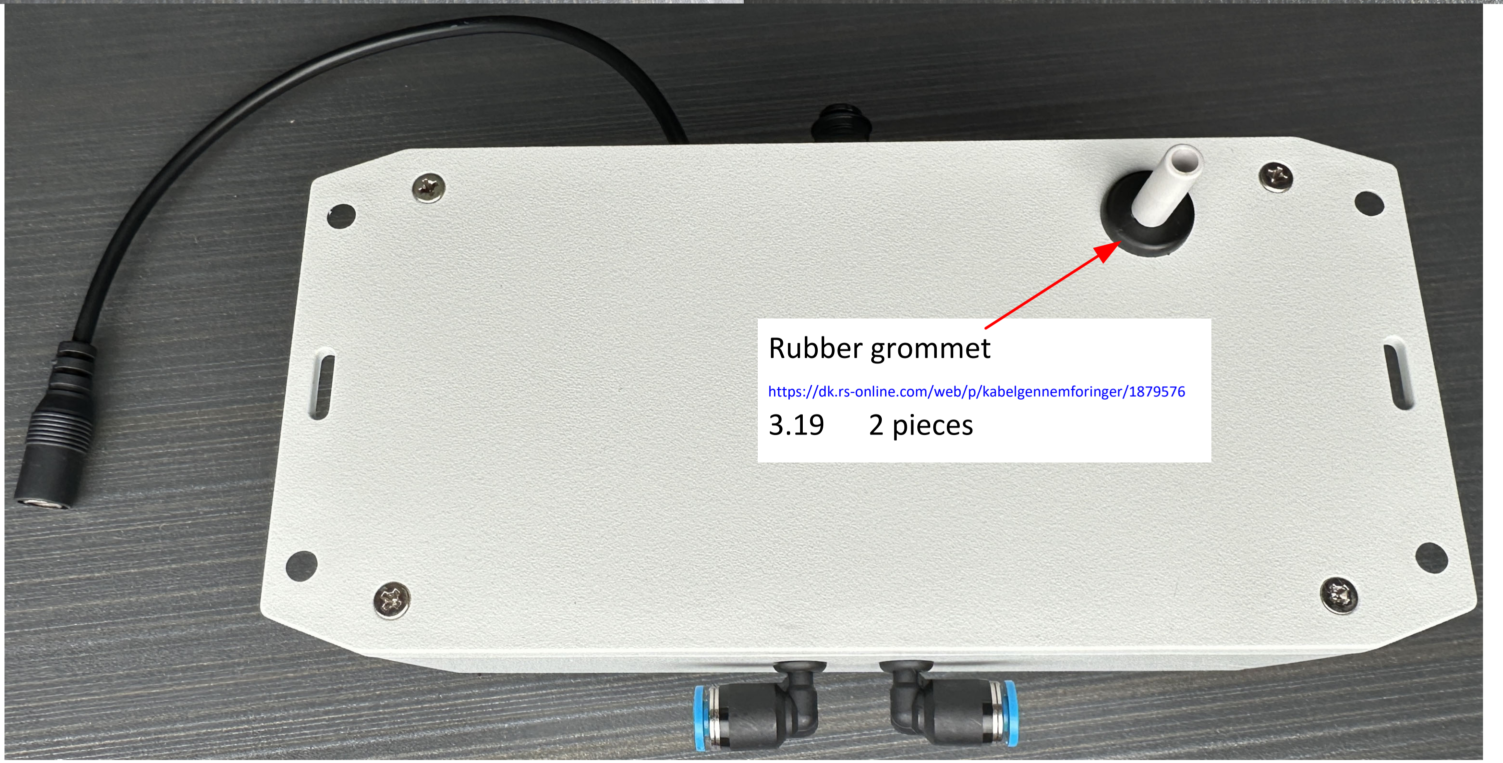

D09 Control box, electrical scheme

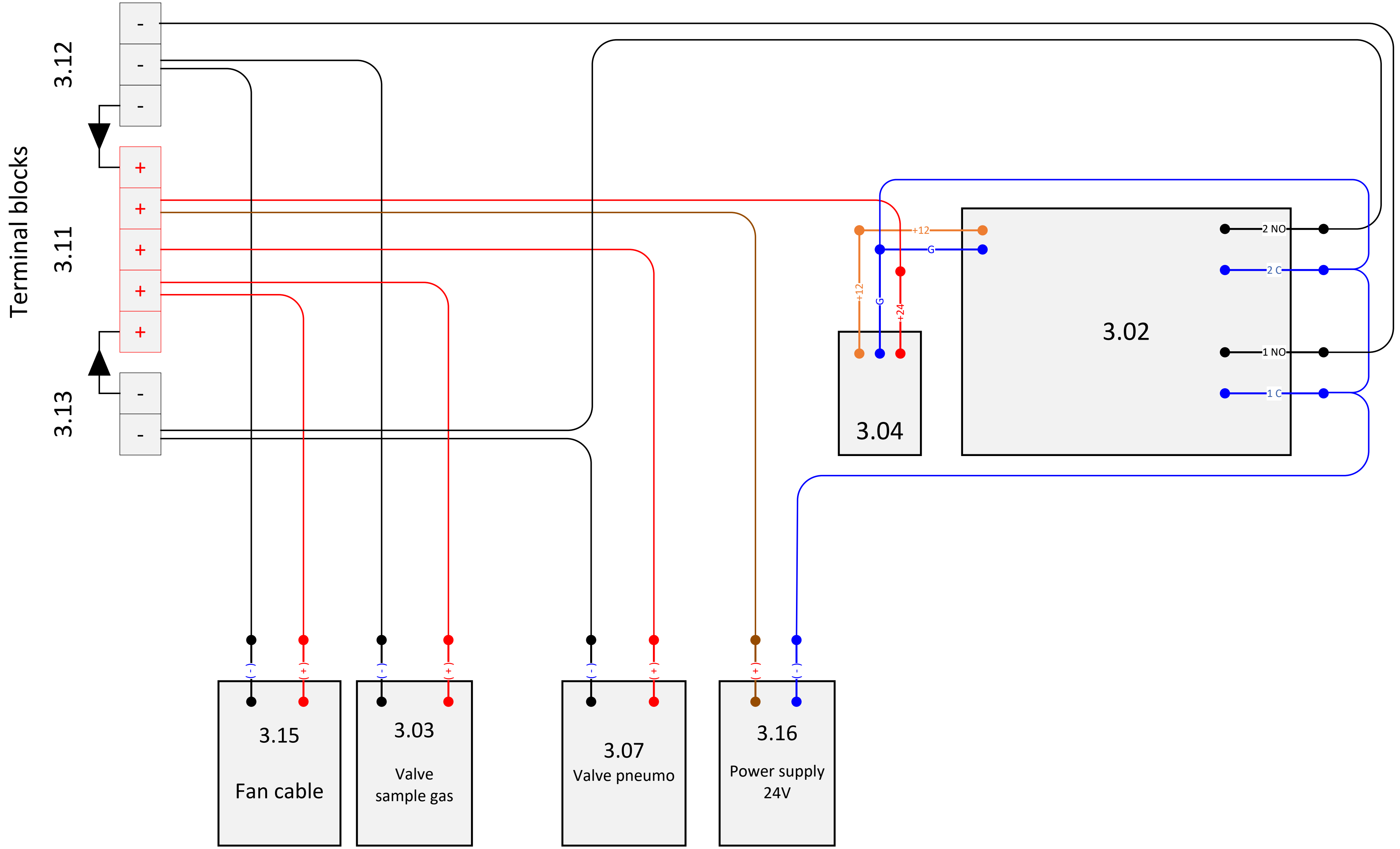

D10 Control box, drill holes

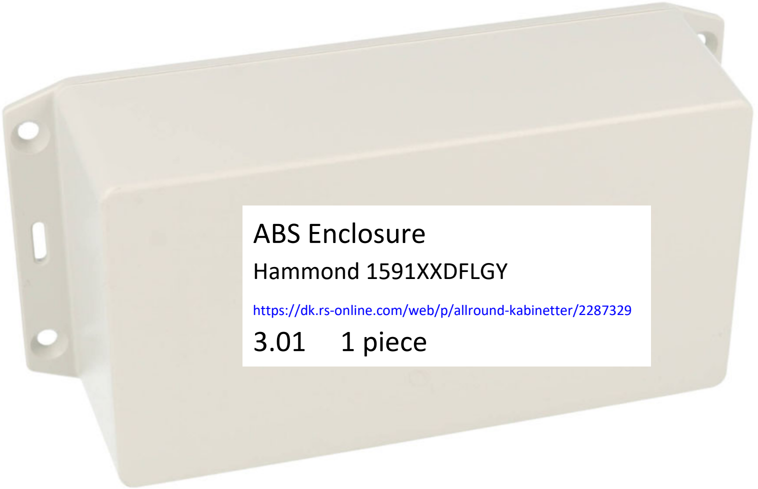

ABS Enclosure

Hammond 1591XXDFLGY

https://dk.rs-online.com/web/p/allround-kabinetter/2287329

3.01 1 piece

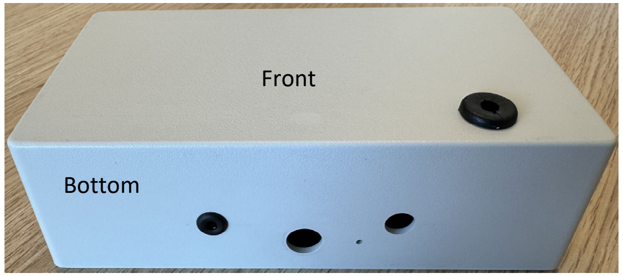

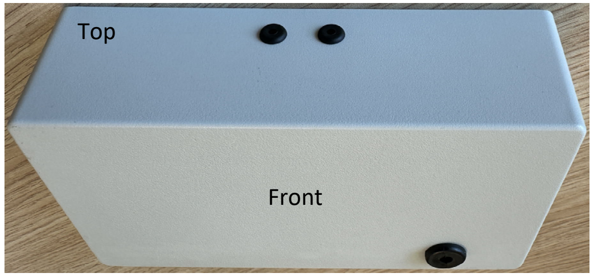

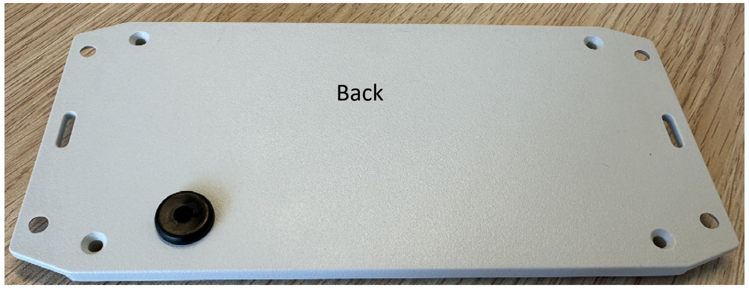

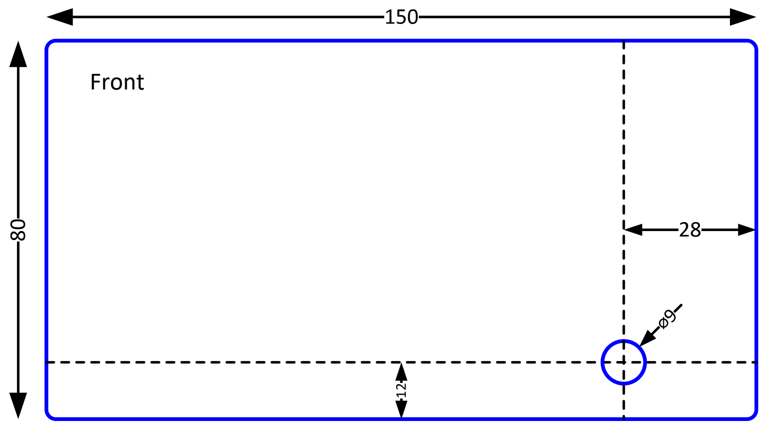

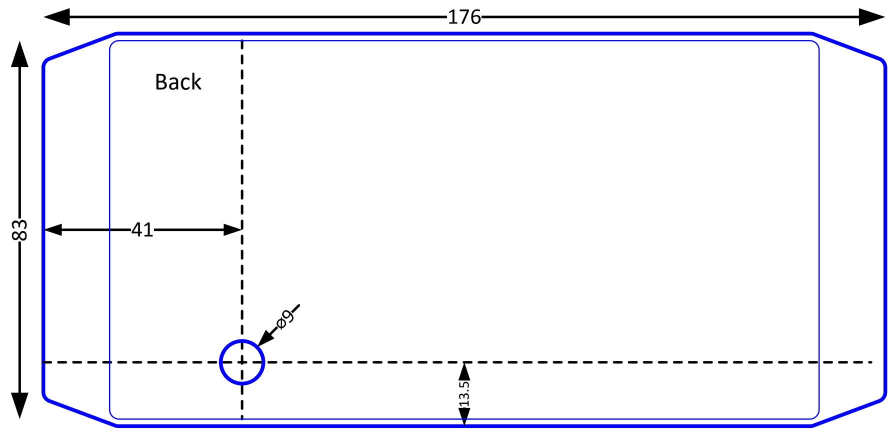

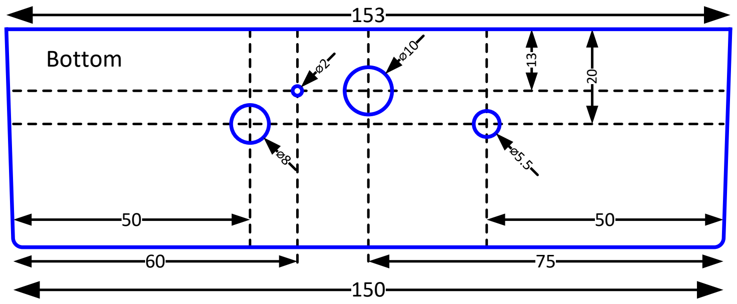

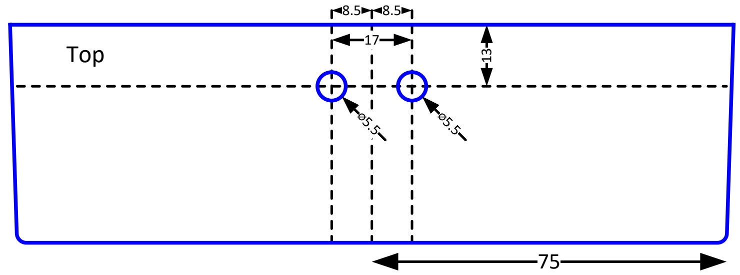

D11 Holes in chamber walls

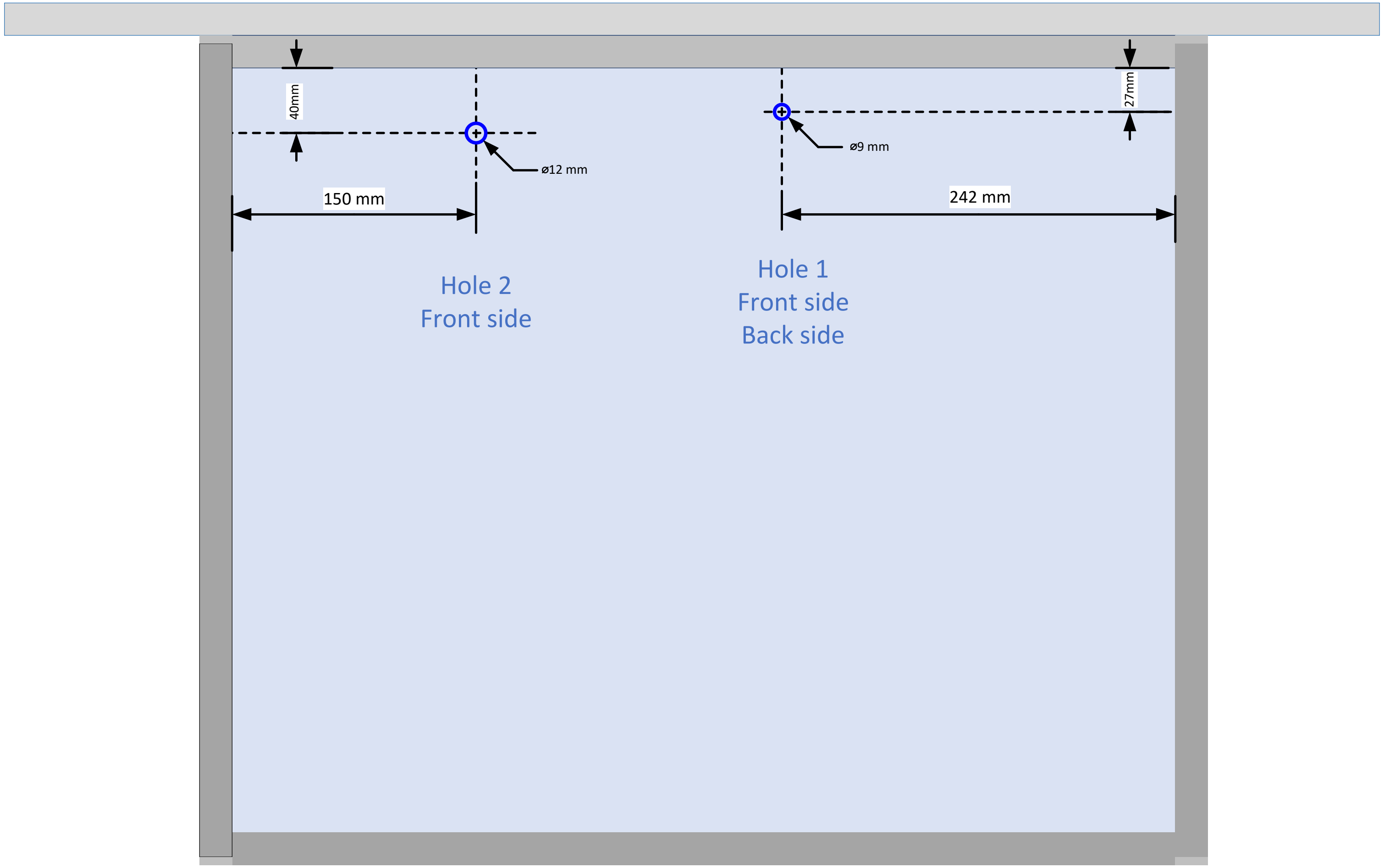

D12 Control box, fan and vent

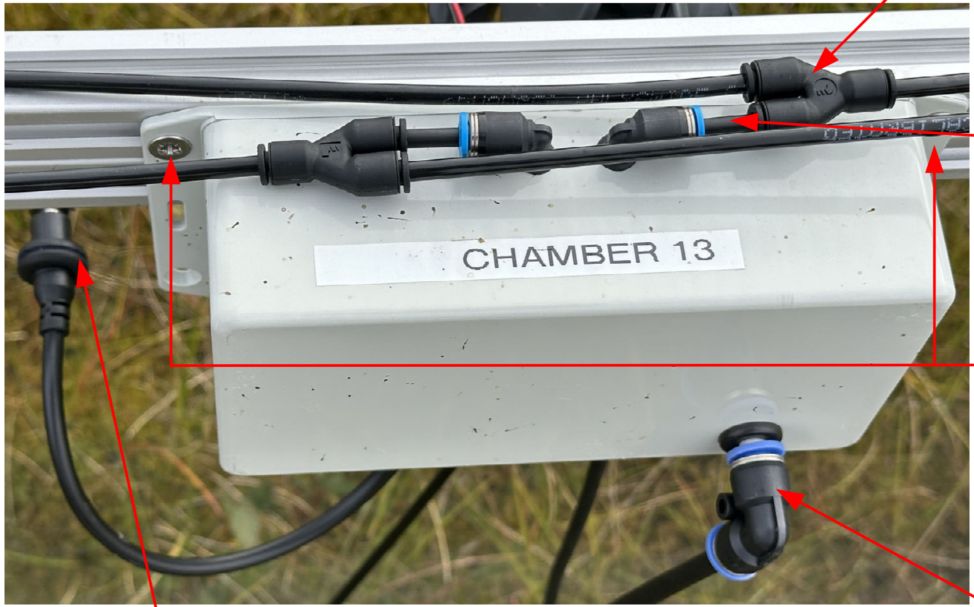

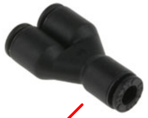

Y adaptor 4 mm

https://dk.rs-online.com/web/p/pneumatik-fittings/0812213

3.24 2 pieces

Straight adaptor 4 mm

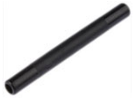

https://dk.rs-online.com/web/p/pneumatik-fittings/2317057

3.22 2 pieces

Screw M5

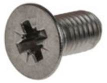

https://dk.rs-online.com/web/p/maskinskruer/9141958

3.27 2 pieces

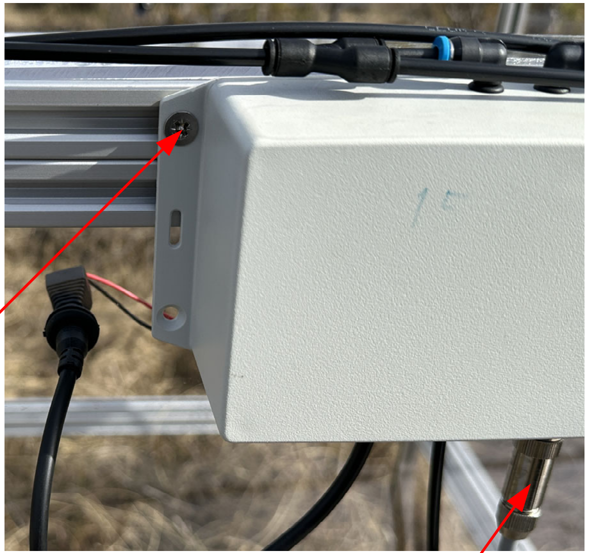

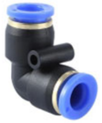

Elbow adaptor 6 mm

https://dk.rs-online.com/web/p/pneumatik-fittings/9160883

3.25 2 pieces

Power connector

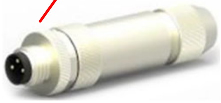

https://dk.rs-online.com/web/p/industrielle-cirkulaere-stik/1345718

3.17 1 piece

Rubber grommet

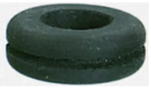

https://dk.rs-online.com/web/p/kabelgennemforinger/1366115

3.26 1 piece

Fan power plug

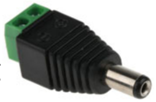

https://dk.rs-online.com/web/p/dc-stik/8104591

3.29 1 piece

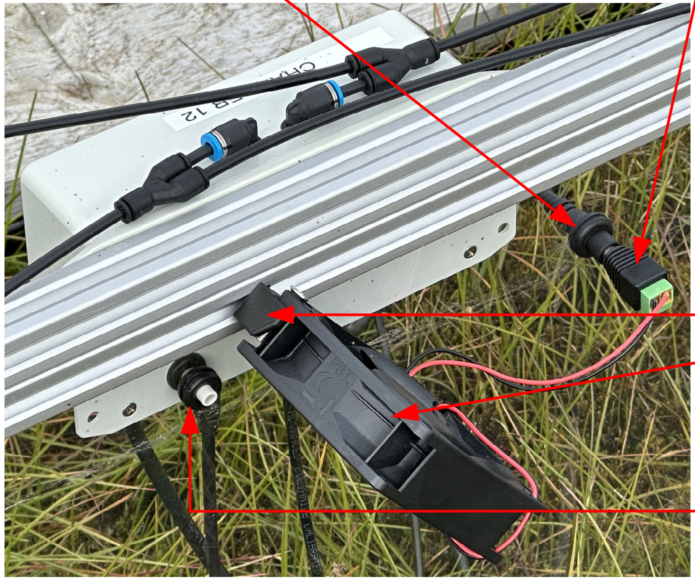

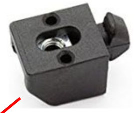

Fan mount 3.30 1 piece

https://www.motedis.dk/en/Universal-block-I-type-slot-5-

Screw M4 3.31 1 piece

https://dk.rs-online.com/web/p/maskinskruer/1583613

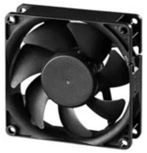

Fan

https://dk.rs-online.com/web/p/aksiale-blaesere/2025439

3.28 1 piece

Rubber grommet

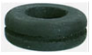

https://dk.rs-online.com/web/p/kabelgennemforinger/1879576

3.19 2 pieces

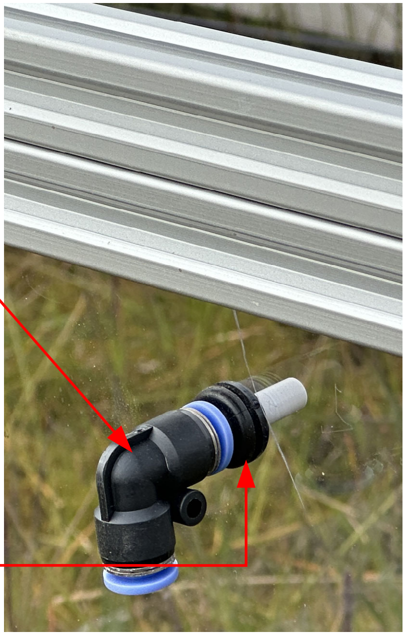

Supplement: Supplementary Data 1 [file mmc1.pdf]
